# The vesicular stomatitis virus matrix protein promotes FACT subunit depletion to suppress the FEAR pathway

**DOI:** 10.1371/journal.ppat.1014430

**Published:** 2026-07-17

**Authors:** Emily A. Rex, Dahee Seo, Aaron Embry, Neal M. Alto, Don B. Gammon

**Affiliations:** Department of Microbiology, University of Texas Southwestern Medical Center, Dallas, Texas, United States of America; University of Tokyo Graduate School of Medicine Faculty of Medicine: Tokyo Daigaku Daigakuin Igakukei Kenkyuka Igakubu, JAPAN

## Abstract

We recently identified the FACT-ETS-1 Antiviral Response (FEAR) pathway as an interferon-independent innate immune response that restricts DNA virus replication and that is antagonized by poxvirus-encoded A51R proteins (Rex *et al.*, 2024, *Nature Microbiology*). The human FEAR pathway is mediated by the FACT complex, consisting of hSpt16 and SSRP1 subunits, that remodels chromatin to activate expression of the antiviral transcription factor, ETS-1. To counter this pathway, poxvirus A51R proteins tether SUMOylated hSpt16 subunits to microtubules to prevent ETS-1 expression. While these observations indicate a role for the FEAR pathway in DNA virus restriction, it was unclear if RNA viruses interact with this pathway. Here, we show that the RNA virus, vesicular stomatitis virus (VSV; *Rhabdoviridae*), is also restricted by the FEAR pathway but has evolved a distinct mechanism to block this antiviral response. Through *in vitro* assays and cell culture studies, we demonstrate that the VSV encoded matrix (M) protein directly interacts with SUMOylated hSpt16 to promote its ubiquitination and proteasome-dependent degradation. Using deletion mapping, we discovered a N-terminal motif in VSV M that is both required for interaction with host hSpt16 proteins and for their proteasomal degradation. This degradation effectively blocks ETS-1 induction and suppresses FEAR pathway activation during infection. Strains encoding mutant M proteins that cannot antagonize the FEAR pathway exhibit replication defects in human cells that can be rescued by hSpt16 or ETS-1 depletion, suggesting FEAR pathway evasion is critical for efficient VSV replication. Moreover, we show that the inability of VSV M to interact with and degrade SUMOylated Spt16 in lepidopteran cells results in an abortive infection, suggesting VSV-Spt16 interactions can influence virus host range. Collectively, our study illustrates that DNA and RNA viruses have independently evolved diverse mechanisms to antagonize SUMOylated host Spt16 proteins, underscoring the physiological importance of the FEAR pathway to antiviral immunity.

## Author summary

Cells have built-in defenses that restrict viral replication during infection. Recently, we discovered one such defense, called the FACT-ETS-1 Antiviral Response (FEAR) pathway. This pathway limits virus replication by turning on the antiviral transcription factor ETS-1. We have previously shown that DNA viruses belonging to the poxvirus family block the FEAR pathway during infection by sequestering Spt16 – a key FACT complex subunit required for ETS-1 expression. Here, we asked whether RNA viruses also interact with the FEAR pathway. Using vesicular stomatitis virus (VSV) as our model RNA virus, we show that VSV inhibits the FEAR pathway but through a different mechanism than poxviruses. We found the VSV-encoded matrix (M) protein both binds, and promotes the degradation of, host Spt16 proteins, which prevents cells from producing ETS-1 and mounting an antiviral response. We identified VSV strains encoding mutations in the M protein that prevent these viruses from blocking the FEAR pathway. These mutants display reduced replication in human cells, suggesting that evasion of the FEAR pathway is critical for efficient VSV replication. Collectively, our results suggest that both DNA and RNA viruses have independently evolved strategies to disable the FEAR pathway, highlighting its important role in protecting cells from viral infection.

## Introduction

The activation of transcription-dependent responses during infection is a fundamental aspect of eukaryotic antiviral immunity [1–3]. In turn, evolutionary pressures on viruses to efficiently replicate in their eukaryotic hosts have driven the acquisition of viral countermeasures to these host responses [4–6]. Perhaps the strongest evidence for the importance of an innate immune response pathway to combating infection is the identification of pathogen-encoded antagonists of that pathway. The fact that virtually all mammalian viruses encode antagonists of the Type I interferon (IFN) response highlights the importance of this antiviral pathway [6–8]. However, relatively little is known regarding IFN-independent antiviral pathways that restrict virus replication and the mechanisms used by viruses to thwart such responses.

We recently reported the discovery of an IFN-independent “FACT-ETS-1 Antiviral Response (FEAR)” pathway that requires the cellular Facilitates Chromatin Transcription (FACT) complex for activation [9]. FACT is an ancient chromatin remodeling factor that is conserved from yeast to humans. In humans, FACT is comprised of human suppressor of ty 16 homologue (hSpt16) and structure specific recognition protein 1 (SSRP1) subunits that regulate cellular gene transcription by assembling and disassembling nucleosomes at specific loci [10,11]. Using vaccinia virus (VV), a large DNA virus belonging to the *Poxviridae* family, we showed that viral infection triggers the FACT-dependent expression of E26 transformation-specific sequence-1 (ETS-1), an antiviral transcription factor, which subsequently promotes VV restriction [9]. ETS-1 expression during infection is dependent upon FACT complexes that contain a specialized, SUMOylated form of hSpt16 (hSpt16^SUMO^) [9]. However, we found the VV-encoded A51R protein to block ETS-1 expression by outcompeting SSRP1 for direct binding to hSpt16^SUMO^ subunits in the cytosol and by tethering hSpt16^SUMO^ to microtubules [9]. VV mutant strains lacking A51R (∆A51R) or encoding A51R mutants unable to bind hSpt16^SUMO^ strongly induce ETS-1 expression and display attenuated replication in human cell culture and in mice [9]. Importantly, ETS-1 induction by ∆A51R infection occurs normally in human cells lacking critical IFN response proteins (e.g., IRF3, IFNAR, STAT1) [9]. Moreover, treatment of cells with recombinant IFN does not induce ETS-1, and knockdown of hSpt16 or ETS-1 does not impair IFN-stimulated gene expression [9]. Collectively, these observations suggest the FEAR pathway is a critical component of eukaryotic antiviral immunity that functions independently of the type I IFN response and that poxvirus A51R proteins can function as FEAR pathway antagonists [9].

Prior to our identification of the FEAR pathway, we had serendipitously discovered that poxvirus A51R proteins could rescue the abortive infection of lepidopteran (moth and butterfly) cells by vesicular stomatitis virus (VSV), a small RNA virus belonging to the *Rhabdoviridae* family [12]. Although VSV is an arbovirus and can be naturally transmitted by blood-feeding dipteran (flies and mosquitoes) insects to mammalian hosts, lepidopterans are unnatural hosts for this virus. Thus, we suspected that A51R proteins may promote productive VSV replication in lepidopteran cells by suppressing an immune response that VSV is incapable of evading in these host cells [12]. Given that A51R proteins are only encoded by vertebrate poxviruses [12], we suspected that these viral proteins are likely immunosuppressive in insect cells because they target antiviral machinery conserved between invertebrate and vertebrate hosts. However, the nature of the host response restricting VSV in these insect cells and suppressed by A51R has remained elusive.

VSV packages an ~ 11 kb negative-sense ssRNA genome encoding five proteins: nucleocapsid (N), phosphoprotein (P), matrix (M), glycoprotein (G), and the large RNA-dependent RNA polymerase (L) [13]. VSV M has been reported to perform several roles during infection, including the induction of cytopathic effects, inhibition of host gene expression, and induction of apoptosis [14–19], and M is thought to be the primary immune evasion protein encoded by VSV [20]. One mechanism by which VSV M inhibits host gene expression involves its interaction with host ribonucleic acid export 1 (Rae1)-nucleoporin 98 (Nup98) mRNA export complexes [17,21,22]. It is thought that VSV M interactions with this complex may block cellular host mRNA export by inhibiting the function of this complex [17], although an alternative model has been proposed wherein VSV M may usurp this complex to facilitate host gene expression cessation [22]. Interestingly, an M protein mutant encoding a methionine 51 deletion (M^∆M51^) or arginine substitution (M^M51R^) is defective in both interacting with Rae1-Nup98 complexes and inhibiting host gene expression [22]. Moreover, this mutant strain is greatly attenuated in its replication, likely due to an inability to inhibit antiviral defenses that depend upon cellular gene expression [16,17,22]. However, it is unclear whether the FEAR pathway contributes to the gene expression-dependent defenses that restrict these VSV mutants.

Understanding innate immune responses that restrict VSV replication is important for many reasons. For one, VSV is associated with outbreaks in livestock in the United States and thus poses a significant economic burden to the agricultural industry [23]. Furthermore, the early development of a reverse genetic system for VSV [24,25], its ability to infect a broad range of host cell types [26], and its inability to cause significant disease in humans [27], have made VSV an important model for understanding fundamental aspects of non-segmented negative-sense ssRNA virus biology.

Building on our discovery of poxvirus A51R proteins as the first DNA virus-encoded FEAR pathway antagonists [9], we used VSV as a model to investigate whether RNA viruses similarly activate and/or antagonize this pathway. Here, we show that the attenuated replication of VSV M^ΔM51/M51R^ strains is at least in part due to their inability to suppress FEAR pathway activation in human cells. In contrast, VSV strains encoding wild-type M proteins effectively suppress the FEAR pathway. We show that wild-type VSV M proteins directly interact with hSpt16 proteins through a conserved N-terminal motif, promoting hSpt16 protein ubiquitination and subsequent proteasome-dependent degradation, ultimately abrogating ETS-1 expression. Importantly, transient expression of wild-type VSV M suppresses infection-induced ETS-1 expression by unrelated viruses, demonstrating that VSV M functions independently and is a bona fide FEAR pathway suppressor. Finally, we show that the well-known host range restriction of VSV in lepidopteran cells [12,28,29] likely results from an inability of VSV M to interact with and promote degradation of SUMOylated Spt16 proteins encoded by these insect hosts. Moreover, our data show that VV A51R relieves this VSV host range restriction by interacting with SUMOylated Spt16 expressed in lepidopteran cells, suggesting that Spt16 proteins have conserved antiviral roles in invertebrates. Collectively, our work illustrates that RNA viruses activate and antagonize the FEAR pathway and identifies the VSV M protein as the first RNA virus-encoded FEAR pathway antagonist.

## Results

### hSpt16 or ETS-1 depletion rescues the replication defect of a VSV^M51R^ mutant

We previously showed that hSpt16 RNAi significantly enhances the replication of VSV in both A549 cells and primary human fibroblasts, suggesting that FACT contributes to VSV restriction [9]. However, we wanted to ask if ETS-1 was also restrictive to VSV replication because it was possible that FACT-mediated VSV restriction is independent of ETS-1 and the FEAR pathway. VSV^∆M51^ or VSV^M51R^ strains are thought to exhibit replication defects due to their inability to block host antiviral gene expression [16,17,20], therefore we wondered whether these replication defects may be related to an inability of these mutants to antagonize the FEAR pathway.

**Fig 1:**
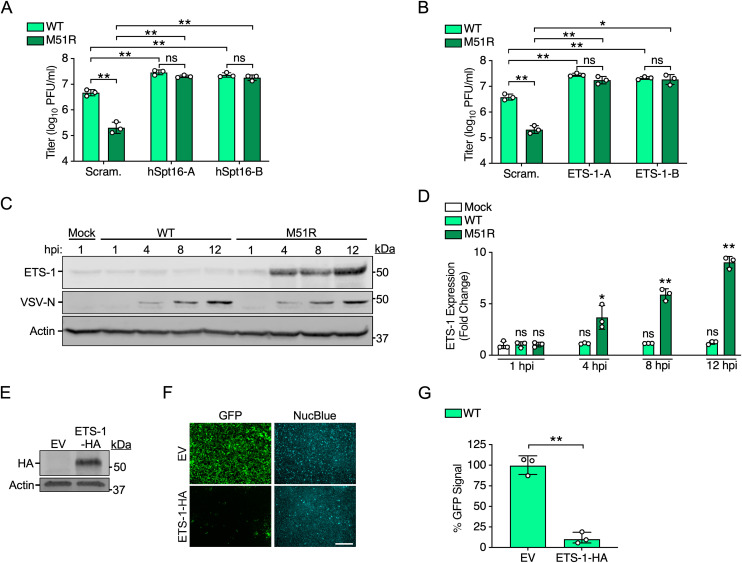
The FEAR pathway contributes to VSV restriction in human cells and is strongly activated by VSV^M51R^-eGFP. (A-B) VSV-eGFP (WT) and VSV^M51R^-eGFP (M51R) titers 24 h post-infection (hpi) (MOI = 0.001) in A549 cells transfected with indicated RNAi treatments using two independent siRNAs for either hSpt16 (A) or ETS-1 (B) knockdown. Scram., scrambled siRNA. Statistical significance in A and B was determined by unpaired two-tailed Student’s t-tests between indicated treatments. (C) Immunoblot (IB) of endogenous ETS-1 in A549 whole cell extract (WCE) after infection with WT or M51R (MOI = 10). (D) Densitometric quantification of ETS-1 from multiple IB experiments as in C. Results of unpaired two-tailed Student’s t-test between ETS-1 levels in mock WCE and infected WCE are shown above each bar graph. (E) IB of U2OS cell WCE transfected with empty vector (EV) or ETS-1-HA constructs 48 h post-transfection. (F) Representative fluorescence microscopy images of U2OS cells transfected as in E and then infected with WT for 16 h. NucBlue was used to mark cell nuclei and to account for cell number. Scale bar = 500μm. (G) Quantification of total GFP signal/field normalized to NucBlue signal for experiments from F. Data are means ± SD; n = 3. Statistical significance was determined by unpaired two-tailed Student’s t-test between indicated treatments. * = P < 0.05; ** = P < 0.01; ns = not significant.

To begin to address these questions, we assessed the replication of recombinant VSV strains encoding eGFP and either wild-type (VSV-eGFP) [30] or M51R mutant M (VSV^M51R^-eGFP) proteins [30] in A549 cells transfected with small interfering RNAs (siRNAs) targeting hSpt16 or ETS-1 [9]. We found hSpt16 or ETS-1 RNAi to significantly enhance VSV-eGFP replication compared to scrambled (control) RNAi conditions, suggesting the FEAR pathway indeed restricts VSV. The VSV^M51R^-eGFP strain exhibited a replication defect compared to VSV-eGFP under control RNAi conditions but strikingly, under either hSpt16 or ETS-1 RNAi conditions, this mutant replicated to titers indistinguishable from VSV-eGFP (Fig 1A and 1B). These results suggest that the FEAR pathway contributes to VSV restriction, however VSV^M51R^-eGFP is more sensitive to restriction by this pathway.

### Wild-type VSV, but not VSV^M51R^, inhibits ETS-1 expression to evade restriction

The results above were reminiscent of our VV studies where we found the ΔA51R strain to exhibit a replication defect that could be rescued by RNAi-mediated depletion of FACT subunits or ETS-1 [9]. We also found this ΔA51R strain to more strongly induce ETS-1 expression than wild-type VV during infection of human cells [9]. Thus, we next used infection time courses and immunoblots (IBs) to determine if ETS-1 levels differ between VSV-eGFP and VSV^M51R^-eGFP infections in A549 cells. Infections were performed under high multiplicity of infection (MOI) conditions to ensure that all cells were infected, thereby allowing the assessment of a single round of infection. Interestingly, while ETS-1 protein levels were not significantly different between mock- and VSV-eGFP-infected cell lysates, VSV^M51R^-eGFP-infected lysates showed significantly elevated ETS-1 by 4 h post-infection (hpi) (Fig 1C and 1D). VSV N was used as a marker for infection. These results indicate that infection with the VSV^M51R^-eGFP mutant induces ETS-1 expression while wild-type (VSV-eGFP) infection does not.

Notably, hSpt16 or ETS-1 depletion still enhanced VSV-eGFP replication by approximately ~1 log (Fig 1A and 1B), despite the absence of detectable ETS-1 induction under the high MOI (10) infection conditions (Fig 1C and 1D) used for immunoblot analysis. One possible explanation is that the viral growth assays were performed under low MOI (0.001), creating conditions in which VSV-eGFP is unable to completely suppress the FEAR pathway, perhaps due to lower expression of its viral antagonist. Thus, VSV-eGFP benefits from prior depletion of Spt16 or ETS-1.

We hypothesized that wild-type VSV suppresses ETS-1 expression to prevent activation of antiviral gene expression programs that require elevated ETS-1 levels. Therefore, we next asked if ETS-1 overexpression prior to infection could suppress VSV-eGFP replication. To do this, we overexpressed an HA-tagged human ETS-1 construct (ETS-1-HA) in cells for 48 h (Fig 1E) and then challenged cells with VSV-eGFP. Using fluorescence microscopy, we quantified GFP signals as a readout for infection and normalized these signals to total cell number by labeling cell nuclei with NucBlue. Compared to cultures transfected with empty vector (control), ETS-1-HA-transfected cells displayed significantly reduced GFP signal (Fig 1F and 1G). Collectively, these data suggest that prior overexpression of ETS-1 in cells limits wild-type VSV-eGFP infection. However, our time course data (Fig 1C and 1D) suggests VSV-eGFP normally evades this restriction by preventing ETS-1 expression throughout infection. In contrast, VSV^M51R^-eGFP fails to prevent cells from inducing ETS-1 during infection (Fig 1C and 1D).

### VSV^M51R^ infection induces ETS-1 expression independently of the IFN response

We showed ΔA51R VV strains to induce ETS-1 expression in the absence of an intact IFN response [9]. Therefore, we wanted to determine if ETS-1 induction by VSV^M51R^-eGFP infection was also independent of the IFN response. Interferon regulatory factor 3 (IRF3) is critical for IFN pathway activation during VSV infection of non-immune cell types [31]. Therefore, we first examined ETS-1 induction in IRF3 knockout A549 cells. No significant differences in ETS-1 induction were found between wild-type and IRF3 knockout cells during VSV^M51R^-eGFP infection (S1A and S1B Fig). We also quantified ETS-1 expression levels in A549 cells lacking STAT1, another key component of the IFN signaling pathway that functions downstream of the IFN-α/β receptor [32,33]. ETS-1 induction by VSV^M51R^-eGFP infection was also unchanged in STAT1 knockout cells compared to control cells (S1C and S1D Fig). Collectively, these data indicate that both DNA viruses (VV) and RNA viruses (VSV) activate ETS-1 expression independently of the IFN response.

### Wild-type VSV, but not VSV^M51R^, promotes hSpt16^SUMO^ depletion to prevent ETS-1 induction during infection

**Fig 2:**
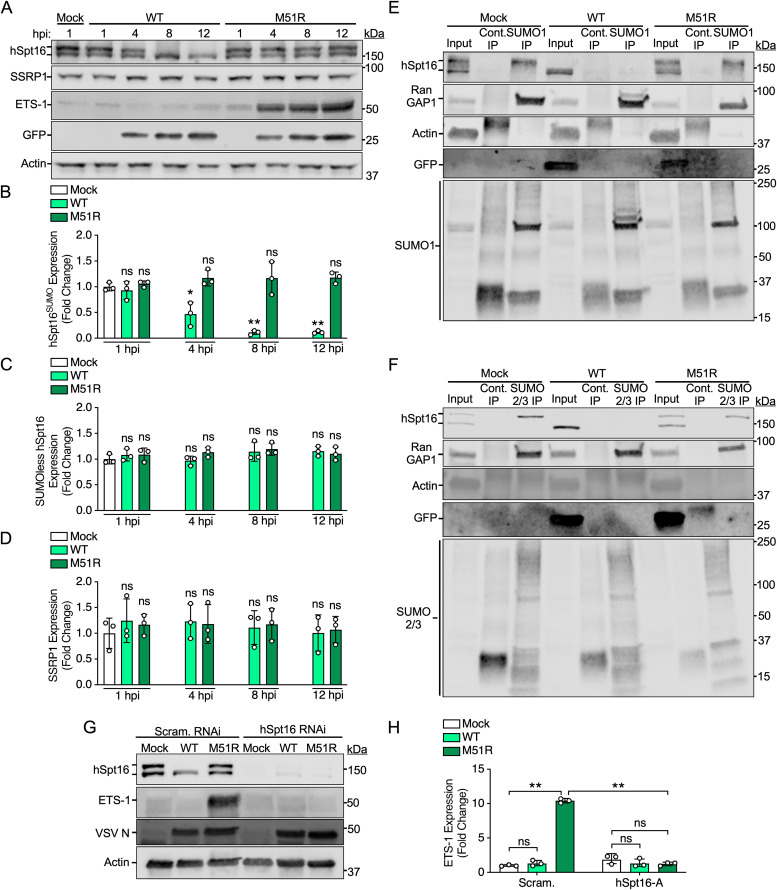
VSV, but not VSV^M51R^, promotes hSpt16^SUMO^ depletion to prevent ETS-1 induction. (A) IB of FACT subunit levels in WCE from A549 cells infected with VSV-eGFP (WT) or VSV^M51R^-eGFP (M51R) for indicated hpi (MOI = 10). (B-D) Densitometric quantification of IB experiments in A for hSpt16^SUMO^ (B) SUMOless hSpt16 (C) or SSRP1 (D) levels. Data in (B-D) are means ± SD; n = 3. Statistical significance was determined by unpaired two-tailed Student’s t-test comparing protein levels in mock WCE to infected WCE, shown above each bar graph. (E-F) IB of immunoprecipitated SUMO-1- (E) or SUMO-2/3- (F) conjugated protein fractions in WCE from mock-, WT-, or M51R-infected A549 cells (MOI = 3) 18 hpi. Cont., control IP. RanGAP1 is a known SUMOylated protein and used as a control for enrichment of SUMOylated fractions [34]. GFP is a marker for infection. (G) IB of endogenous ETS-1 and hSpt16 levels and in WCE of scrambled or hSpt16 siRNA-treated A549 cells 8 hpi with indicated strains (MOI = 10). (H) Densitometric quantification of IB experiments in G for ETS-1. Data are means ± SD; n = 3. Statistical significance was determined by unpaired two-tailed Student’s t-test between indicated treatments. ** = P < 0.01, or ns = not significant.

During FEAR pathway activation, FACT complexes comprised of hSpt16^SUMO^ and SSRP1 are required to induce downstream ETS-1 expression [9]. Therefore, we hypothesized that VSV-eGFP may suppress ETS-1 expression by altering FACT subunit levels. Using high MOI infection time courses and IBs, we determined if hSpt16^SUMO^ or SSRP1 levels differ between VSV-eGFP and VSV^M51R^-eGFP infections. It is important to note that both hSpt16^SUMO^ and SUMOless hSpt16 forms can be resolved from one another by SDS-PAGE and simultaneously detected via IB with hSpt16 antibodies (Abs) [9]. Strikingly, our IBs revealed a clear and significant reduction of hSpt16^SUMO^ (upper band in Fig 2A) levels in VSV-eGFP-infected lysates compared to mock-infected lysates by 4 hpi, and hSpt16^SUMO^ levels became virtually undetectable by 8 hpi. In contrast, hSpt16^SUMO^ levels remained unchanged throughout the entire VSV^M51R^-eGFP infection time course (Fig 2A and 2B). Interestingly, SUMOless hSpt16 (lower band in Fig 2A) or SSRP1 levels did not significantly change during infection with either virus (Fig 2A, 2C and 2D), suggesting that VSV infection leads to the specific depletion of hSpt16^SUMO^ subunits. When comparing the effect of MOI on hSpt16^SUMO^ depletion during VSV-eGFP infection, we noticed that complete depletion of hSpt16^SUMO^ levels occurred earlier (by 4 hpi) when using higher MOIs (e.g., 10 or 30) versus during lower MOI (e.g., 3) infections. However, VSV^M51R^-eGFP could not deplete hSpt16^SUMO^ at any MOI or time point (Figs 2A and S2). Together, these findings suggest that VSV-eGFP depletes hSpt16^SUMO^ during infection, and that the loss of hSpt16^SUMO^ correlates with the absence of ETS-1 induction.

Next, we wanted to determine if the phenotypes we observed using the A549 cell line would also be observed in primary human cells. Thus, we used neonatal human dermal fibroblasts (NHDF), which we have previously shown to restrict viral replication in a hSpt16-dependent manner [9]. Consistent with a role for the FEAR pathway in restricting viral replication in these cells, hSpt16 or ETS-1 RNAi promoted VSV replication. Moreover, the replication defect of VSV^M51R^-eGFP was fully complemented during conditions of hSpt16 or ETS-1 knockdown (S3A and S3B Fig). In addition, as with A549 cells, ETS-1 levels were strongly induced by VSV^M51R^-eGFP, but not by VSV-eGFP, in NHDF cells (S3C and S3D Fig). Furthermore, only VSV-eGFP promoted hSpt16^SUMO^ depletion in these primary cells (S3C and S3E Fig). As with A549 cells, we did not observe infection induced changes in SUMOless hSpt16 or SSRP1 levels compared to mock-infected lysates (S3C, S3F and S3G Fig). Collectively, these data suggest that the FEAR pathway also restricts VSV in primary cells and that VSV-eGFP can also deplete hSpt16^SUMO^ and block ETS-1 induction in these cells.

To determine if loss of hSpt16^SUMO^ levels during VSV-eGFP infection was due to a global down-regulation of intracellular SUMOylation, we used Abs against SUMO-1 or SUMO-2/3 to immunoprecipitate (IP) SUMOylated protein fractions from mock-, VSV-eGFP-, or VSV^M51R^-eGFP-infected lysates [9]. As expected, hSpt16^SUMO^ was specifically depleted in VSV-eGFP-infected lysates, while present in mock- and VSV^M51R^-eGFP-infected lysates. Consistent with our prior study [9], hSpt16^SUMO^ bands were observed in either SUMO-1 or SUMO-2/3 IPs, indicating that hSpt16 can be SUMOylated with SUMO-1 or SUMO-2/3 proteins. Importantly, no overt reductions in either total SUMOylation banding patterns or levels of SUMOylated-RanGAP1 (an abundant, SUMOylated protein [34]) were observed in VSV-eGFP versus VSV^M51R^-eGFP infections (Fig 2E and 2F). These data suggest that VSV-eGFP promotes depletion of hSpt16 SUMOylated with either SUMO-1 or SUMO-2/3, and that the specific loss of hSpt16^SUMO^ levels during VSV-eGFP infection is not due to a generalized decrease in intracellular SUMOylation.

Finally, because hSpt16^SUMO^ is required for ETS-1 induction during VV infection [9], we wanted to determine if hSpt16 knockdown prior to infection would inhibit ETS-1 induction by VSV^M51R^-eGFP. Consistent with our VV studies, hSpt16 RNAi abrogated ETS-1 induction after VSV^M51R^-eGFP infection (Fig 2G and 2H). Collectively, these data indicate that the inability of VSV^M51R^-eGFP to deplete hSpt16^SUMO^ results in FEAR pathway activation, robust ETS-1 expression, and subsequent restriction of viral replication.

### VSV M promotes depletion of cytosolic hSpt16^SUMO^ subunits independently of other VSV proteins

**Fig 3:**
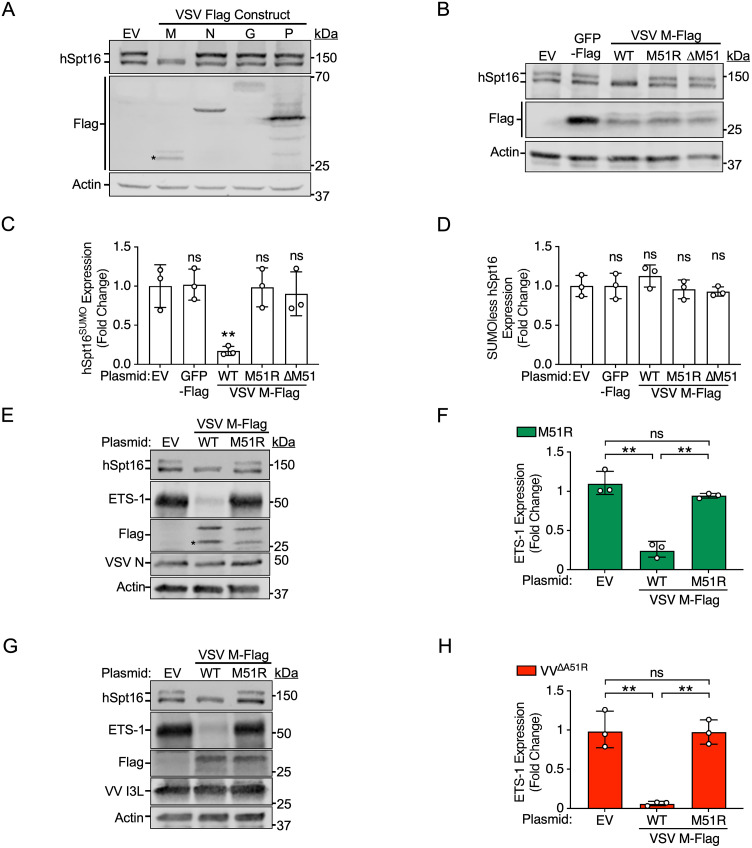
VSV M promotes hSpt16^SUMO^ depletion independently of other VSV proteins and blocks ETS-1 expression. (A) IB of endogenous hSpt16 in 293T whole cell extract (WCE) transfected with indicated Flag-tagged VSV protein expression constructs for 24 h. Empty vector, EV; Matrix, M; Nucleocapsid, N; Glycoprotein, G; Phosphoprotein, P. Asterisks indicate probable VSV M degradation products. (B) IB of endogenous hSpt16 in 293T WCE transfected with indicated VSV M expression constructs 24 h post-transfection. EV and Flag-GFP constructs were negative controls. (C-D) Densitometric quantification of IB experiments in B for hSpt16^SUMO^ (C) and SUMOless hSpt16 (D). Data are means ± SD; n = 3. Statistical significance was determined by unpaired two-tailed Student’s t-test comparing protein levels in EV to indicated transfection treatments, shown above each bar graph. ** = P < 0.01, ns = not significant. (E) IB of endogenous ETS-1 in U2OS WCE after transfection with indicated expression constructs for 24 h and then infected with VSV^M51R^-eGFP (M51R) (MOI = 10) for 8 h. (F) Densitometric quantification of IB experiments in E for ETS-1. (G) IB of endogenous ETS-1 in U2OS WCE after transfection with indicated expression constructs for 24 h and then infected with ΔA51R VV strain (MOI = 10) for 8 h. (H) Densitometric quantification of IB experiments in G for ETS-1. In F and H, data are means ± SD; n = 3. Statistical significance was determined by unpaired two-tailed Student’s t-test between indicated treatments. ** = P < 0.01, ns = not significant.

Given that the only difference between VSV-eGFP and VSV^M51R^-eGFP is the M51R substitution in VSV M, we hypothesized that this viral factor was responsible for hSpt16^SUMO^ depletion. Consistent with this, a VSV^∆M51^ strain expressing GFP (VSV^∆M51^-GFP [35]) was also unable to deplete hSpt16^SUMO^ levels and strongly induced ETS-1 expression when compared to its wild-type VSV-GFP control strain (S4 Fig). However, we still conducted a screen of 4 of the 5 VSV-encoded proteins to determine if any of these viral factors could reduce hSpt16^SUMO^ levels when expressed from plasmids in uninfected human cells. The VSV L protein, encoding the viral RNA polymerase, was excluded from this screen as it seemed unlikely to be involved in immune evasion. Consistent with our hypothesis, only expression of Flag-tagged VSV M (VSV M-Flag) resulted in hSpt16^SUMO^ depletion (Fig 3A). Expression of either VSV M^M51R^-Flag or VSV M^∆M51^-Flag mutant constructs failed to deplete hSpt16^SUMO^ levels (Fig 3B and 3C). Moreover, these did not alter SUMOless hSpt16 levels (Fig 3B and 3D), consistent with our infection results. These data indicate that wild-type VSV M, but not M^M51R^ or M^ΔM51^, can promote hSpt16^SUMO^ depletion in the absence of other VSV proteins.

Given that VV A51R blocks the FEAR pathway by binding to cytosolic hSpt16^SUMO^ subunits [9], we next asked if VSV M also targets cytosolic hSpt16^SUMO^ subunits. To do this, we co-expressed VSV M-Flag constructs with either a HA-tagged wild-type (HA-hSpt16) or nuclear localization sequence (NLS)-deleted (HA-hSpt16^∆NLS^) hSpt16 construct we reported previously [9]. Importantly, we confirmed that HA-hSpt16 displayed both cytosolic and nuclear localization while HA-hSpt16^∆NLS^ was restricted to the cytosol, as expected (S5A Fig). Upon co-transfection with VSV M-Flag, the SUMOylated forms of both HA-hSpt16 and HA-hSpt16^∆NLS^ were depleted (S5B Fig), indicating VSV M can promote depletion of cytosolic hSpt16^SUMO^ subunits.

### VSV M proteins from the Indiana and New Jersey strains deplete hSpt16^SUMO^ and block infection-induced ETS-1 expression

We next examined if plasmid-based expression of VSV M-Flag was sufficient to block ETS-1 induction by either VSV^M51R^-eGFP (Fig 3E and 3F) or ΔA51R VV (Fig 3G and 3H) infection. Prior expression of VSV M-Flag, but not VSV M^M51R^-Flag, was able to suppress ETS-1 induction by either of mutant virus (Fig 3E-3H). Given that the experiments above utilized the VSV M protein derived from the Indiana serotype (VSV-IN), we asked whether M from the New Jersey serotype (VSV-NJ) [36] [which shares ~59.8% amino acid sequence identity with VSV-IN (S6A Fig)] depleted hSpt16^SUMO^. Like VSV-IN M, the M protein from VSV-NJ also depleted hSpt16^SUMO^ and inhibited ETS-1 induction when expressed transiently in cells (S6B–S6D Fig). The ability of VSV M proteins to block ΔA51R-induced ETS-1 expression demonstrates that these viral factors are bona fide FEAR pathway antagonists that function independently of other VSV proteins. Collectively, our prior [9] and current findings suggest that VV and VSV have independently evolved unique strategies to inhibit FACT-dependent ETS-1 expression by either tethering hSpt16^SUMO^ to microtubules (VV A51R) or by promoting hSpt16^SUMO^ degradation (VSV M), illustrating that FEAR pathway suppression is important for both DNA and RNA viruses.

### Rae1-Nup98 interaction with VSV M does not contribute to hSpt16^SUMO^ depletion

VSV M is thought to inhibit host gene expression by interacting with Rae1, a component of the cellular Rae1-Nup98 protein complex that exports mRNA from the nucleus [17], although the exact mechanism is controversial [17,22]. In one proposed model, VSV M binds Rae1 to inhibit Rae1-Nup98-mediated mRNA export from the nucleus while an alternative model suggests VSV M usurps Rae1-Nup98 complexes to inhibit cellular gene transcription [17,22]. Notably, VSV M^M51R^ or M^ΔM51^ mutants cannot interact with Rae1 [17]. Despite the unresolved nature of VSV M-Rae1 interaction models, we wanted to determine if Rae1 or Nup98 depletion would affect hSpt16^SUMO^ levels. Thus, we tested if Rae1 or Nup98 RNAi altered hSpt16^SUMO^ levels in mock-infected cells or if their depletion inhibited the ability of VSV-eGFP to reduce hSpt16^SUMO^ levels during infection. In either case, we observed no effect of Rae1 or Nup98 depletion on hSpt16^SUMO^ levels (S7A and S7B Fig). This suggests that VSV M antagonism of the FEAR pathway cannot be explained by its interaction with Rae1-Nup98 complexes.

### VSV M-mediated hSpt16^SUMO^ depletion requires the ubiquitin-proteasome system (UPS)

**Fig 4:**
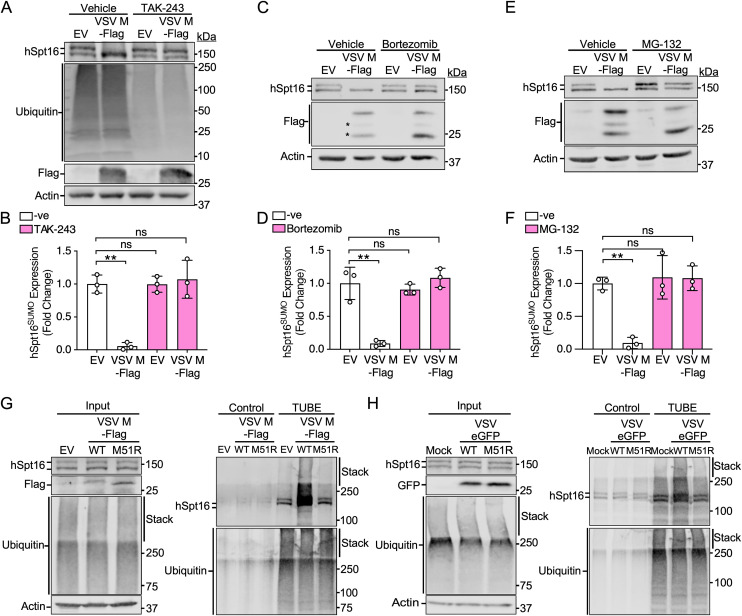
VSV M, but not VSV M^M51R^, promotes hSpt16 ubiquitination, leading to the proteasomal-degradation of hSpt16^SUMO^. (A) IB of endogenous hSpt16 in U2OS whole cell extract (WCE) after transfection with empty vector (EV) or VSV M-Flag. Where indicated, 1 μM TAK-243 was added at 6 h post-transfection. Vehicle (DMSO) treatments served as negative controls. WCE were prepared 24 h post-transfection. (B) Densitometric quantification of IB experiments in A for hSpt16^SUMO^. (C) IB of endogenous hSpt16 in U2OS WCE after transfection with EV or VSV M-Flag. Where indicated, 25 nM bortezomib was added at 6 h post-transfection. Vehicle (DMSO) treatments served as negative controls. WCE were prepared 24 h post-transfection. (D) Densitometric quantification of IB experiments in C for hSpt16^SUMO^. (E) IB of endogenous hSpt16 in U2OS WCE after transfection with EV or VSV M-Flag. Where indicated, 40 μM MG-132 was added at 6 h post-transfection. Vehicle (DMSO) treatments served as negative controls. WCE were prepared 24 h post-transfection. (F) Densitometric quantification of IB experiments in E for hSpt16^SUMO^. (G) IB of TUBE pulldowns from 293T cells transfected with EV, VSV M or M^M51R^-Flag for 24 h. Bortezomib was added to all cultures at 6 h. (H) IB of TUBE pulldowns from mock-, VSV-eGFP (WT) or VSV^M51R^-eGFP (M51R) infected A549 cells for 24 h (MOI = 10). Bortezomib was added to all cultures at 6 hpi. In B, D and F, data are means ± SD; n = 3. Statistical significance was determined by unpaired two-tailed Student’s t-test between indicated treatments. ** = P < 0.01, ns = not significant.

Most proteins in the cell are turned over by the UPS [37]. Therefore, we first wanted to determine if VSV M-mediated hSpt16^SUMO^ depletion required ubiquitination. We expressed VSV M-Flag in human cells in the presence or absence of a ubiquitin activating enzyme inhibitor, TAK-243 [38], which globally blocks ubiquitination. We found TAK-243 treatment to prevent VSV M-mediated depletion of hSpt16^SUMO^, suggesting ubiquitination is required for hSpt16^SUMO^ loss (Fig 4A and 4B). We then determined whether VSV M promotes proteasome-dependent degradation of hSpt16^SUMO^ by chemically inhibiting the proteasome using bortezomib or MG-132. hSpt16^SUMO^ depletion was blocked in the presence of either proteasome inhibitor (Fig 4C–4F), suggesting VSV M promotes the proteasome-dependent degradation of hSpt16^SUMO^.

### VSV M, but not VSV M^M51R^, promotes ubiquitination of hSpt16 proteins

Given that VSV M promotes proteasome-dependent degradation of hSpt16^SUMO^, we asked whether hSpt16 was differentially ubiquitinated during transient expression of VSV M or M^M51R^. Importantly, we included bortezomib in our cultures to prevent hSpt16^SUMO^ degradation during VSV M expression. Cells were harvested under denaturing buffer conditions then incubated with Tandem Ubiquitin Binding Entities (TUBEs), which are beads conjugated to pan-ubiquitin-binding domains designed to enrich for ubiquitinated proteins [39]. The ubiquitination pattern of hSpt16 was then assessed via IB. Although we did not observe overt differences in ubiquitination patterns in our lysate inputs across transfection treatments, after TUBE-based enrichment of ubiquitinated protein fractions, there was a clear increase in high molecular weight hSpt16 protein species in wild-type VSV M-Flag-transfected lysates, indicative of ubiquitinated forms of hSpt16. In contrast, these higher molecular weight hSpt16 species were largely absent in empty vector or VSV M^M51R^-Flag transfected lysates (Fig 4G). These data suggest that wild-type VSV M promotes hyper-ubiquitination of hSpt16 in the absence of other VSV proteins. Similar hSpt16 hyper-ubiquitination patterns were observed during VSV-eGFP, but not VSV M^M51R^-eGFP, infection suggesting VSV M also promotes hSpt16 ubiquitination in the context of infection (Fig 4H).

### VSV M directly interacts with hSpt16 proteins

**Fig 5:**
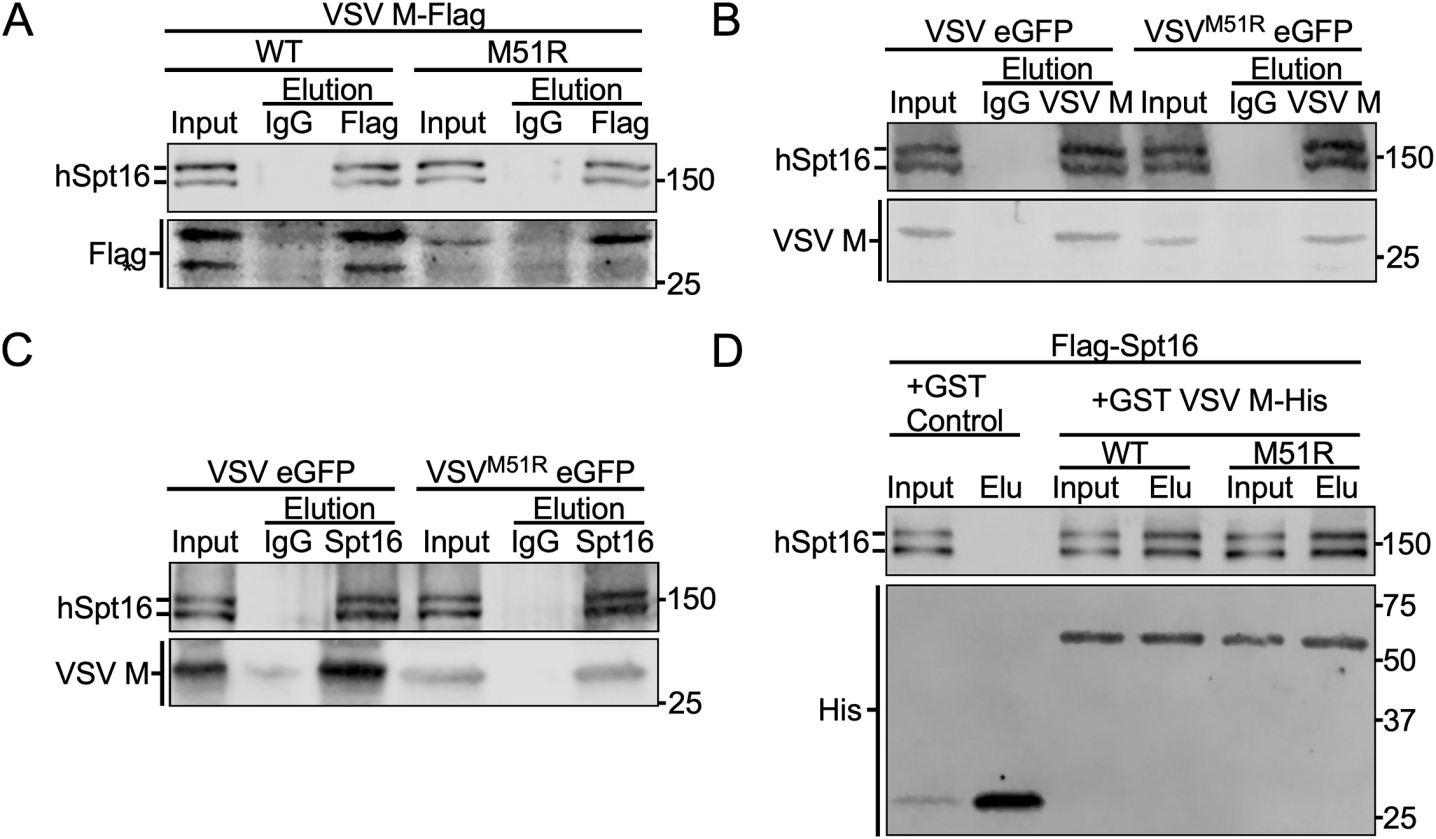
VSV M interacts with hSpt16. (A) Representative IB of VSV M or M^M51R^-Flag proteins and hSpt16 after immunoprecipitation with Flag or isotype control immunoglobulin (IgG) Abs from transfected 293T whole cell extract (WCE) 24 h post-transfection. Bortezomib was added to all cultures at 6 h post-transfection. (B) Representative IB of VSV-eGFP (WT) or VSV^M51R^-eGFP (M51R) M proteins and hSpt16 after immunoprecipitation with VSV M or control IgG Abs from infected A549 lysates 24 hpi (MOI = 10). Bortezomib was added to all cultures at 6 hpi. (C) Representative IB of hSpt16 and WT or M51R M proteins after immunoprecipitation with hSpt16 or control IgG Abs from WT or M51R infected A549 lysates 24 hpi (MOI = 10). Bortezomib was added to all cultures at 6 hpi. (D) Representative IB of *in vitro* GST pulldown of purified Flag-hSpt16 proteins incubated with purified GST-His, GST-VSV M-His, or GST-VSV M^M51R^-His proteins using glutathione beads.

Since VSV M promotes hSpt16 ubiquitination, yet only induces hSpt16^SUMO^ (but not SUMOless hSpt16) degradation, we hypothesized that VSV M, but not the M^M51R^ mutant, interacts with hSpt16^SUMO^ for targeted degradation. To test this hypothesis, we performed coimmunoprecipitation (Co-IP) assays using Flag Abs from lysates transiently expressing VSV M- or M^M51R^-Flag in the presence of bortezomib. Surprisingly, we observed endogenous hSpt16^SUMO^ and SUMOless hSpt16 to both Co-IP with VSV M (Fig 5A). This was unexpected because VSV M only promotes the degradation of hSpt16^SUMO^, yet these data suggest that VSV M interacts with both hSpt16 and hSpt16^SUMO^ proteins. Furthermore, VSV M^M51R^ also co-immunoprecipitated with both forms of hSpt16 (Fig 5A). This was also unexpected since VSV M^M51R^ is unable to promote hSpt16^SUMO^ degradation (Fig 3B and 3C). To validate these results in the context of infection, we performed additional Co-IPs from VSV-eGFP- or VSV^M51R^-eGFP-infected lysates using VSV M Abs. Similar to when VSV M-Flag was expressed in uninfected cells, we found both hSpt16 forms to Co-IP with VSV M and VSV M^M51R^ in infected cells (Fig 5B). We performed reciprocal Co-IPs using Abs against endogenous hSpt16 and again observed VSV M and M^M51R^ to Co-IP with both forms of hSpt16 during infection (Fig 5C). Together these data suggest that VSV M proteins interact with both SUMOylated and SUMOless hSpt16 proteins.

Next, we wanted to determine if the VSV M and hSpt16/hSpt16^SUMO^ interaction is direct. To do this, we used *in vitro* pulldown experiments with purified Flag-tagged hSpt16/hSpt16^SUMO^ [9] and purified VSV M proteins encoding an N-terminal GST tag and a C-terminal His tag (GST-VSV M-His). GST encoding a C-terminal His tag (GST-His) was used as a negative control protein. We incubated purified Flag-hSpt16/hSpt16^SUMO^ with either GST-His, GST-VSV M-His, or GST-VSV M^M51R^-His, then pulled down GST-fused proteins using glutathione beads. GST-His failed to pull down either Flag-hSpt16 or hSpt16^SUMO^, however, both GST-VSV M-His and -M^M51R^-His interacted with both forms of Flag-hSpt16 (Fig 5D), consistent with our Co-IP data (Fig 5A–5C). Collectively, this suggests that VSV M and M^M51R^ directly bind to both SUMOless and SUMOylated hSpt16, and that the M51R mutation does not affect hSpt16 binding.

### The N-terminus of VSV M is required for interaction with hSpt16 proteins and FEAR pathway suppression

**Fig 6:**
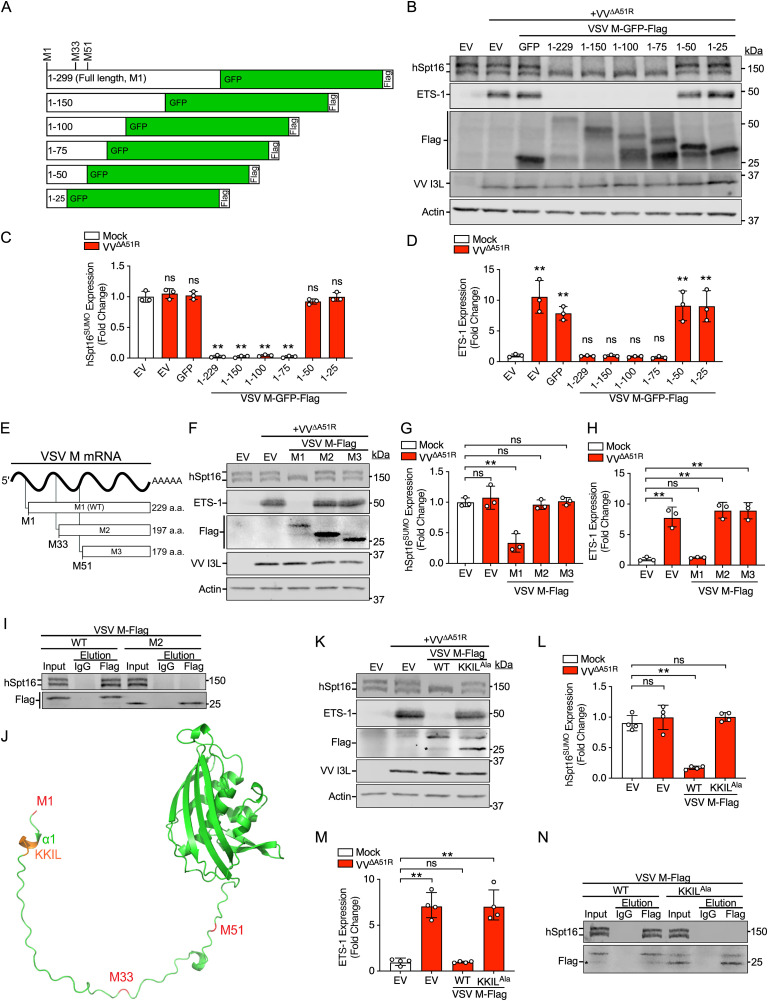
N-termini of VSV M mediates hSpt16 interaction and promotes hSpt16^SUMO^ degradation. (A) Diagram of N-terminal VSV M-GFP-Flag fusion constructs. (B) IB of U2OS whole cell extract (WCE) 24 h post-transfection with indicated VSV M-GFP-Flag constructs infected with VV ∆A51R (VV^∆A51R^) to assess ETS-1 induction. I3L is used as a marker for VV infection. (C-D) Densitometric quantification of hSpt16^SUMO^ (C) or ETS-1 (D) from multiple IB experiments as in B. (E) Diagram of differentially expressed VSV M proteins from VSV M mRNA. Full length a.a. 1-229, M1; a.a. 33-229, M2; a.a. 51-229, M3 showing alternative translation start sites. (F) IB of endogenous hSpt16 in 293T lysates transfected with indicated VSV M expression constructs from E 24 h post-transfection then infected with VV ∆A51R (MOI = 10) for 8 h. EV constructs served as negative controls. (G-H) Densitometric quantification of IB experiments in F for hSpt16^SUMO^ (G) and ETS-1 (H). (I) Representative IB of VSV M1- or M2-Flag proteins and hSpt16 after immunoprecipitation with Flag or isotype control immunoglobulin (IgG) Abs from transfected 293T lysates 24 h post-transfection. Bortezomib was added to all cultures at 6 h post-transfection. (J) Alphafold3-generated structure of full-length (a.a. 1-229) VSV M (strain Indiana). M1, M33 and M51 are shown in red and represents the first methionine of M1, M2, and M3 forms of VSV M, respectively. Alpha-helix 1 (α1) noted in green. KKIL motif highlighted in orange. (K) Representative IB of endogenous hSpt16 and ETS-1 in U2OS WCE after transfection with indicated expression constructs for 24 h and then infected with ΔA51R VV strain (MOI = 10) for 8 h. (L-M) Densitometric quantification of IB experiments in K for hSpt16^SUMO^ (L) and ETS-1 (M). (N) Representative IB of VSV M or KKIL^Ala^-Flag proteins and hSpt16 after immunoprecipitation with Flag or isotype control immunoglobulin (IgG) Abs from transfected 293T lysates 24 h post-transfection. Bortezomib was added to all cultures at 6 h post-transfection. For data in C-D, G-H, and L-M, data are means ± SD; n = 3 and statistical significance was determined by unpaired two-tailed Student’s t-test between indicated treatments. ** = P < 0.01, ns = not significant.

Since VSV M^M51R^ does not affect Spt16 binding, yet fails to promote hSpt16^SUMO^ degradation, we hypothesized that residues beyond M51 are required for hSpt16 interaction and subsequent hSpt16^SUMO^ degradation. To identify VSV M regions potentially involved in hSpt16 binding and hSpt16^SUMO^ degradation, we generated a series of constructs with increasing N-terminal lengths of VSV M fused to GFP-Flag (Fig 6A). We then assessed the ability of these constructs to promote hSpt16^SUMO^ degradation and suppress ETS-1 induction. Notably, VSV M fragments containing the first 75 residues retained the ability to promote hSpt16^SUMO^ depletion (Fig 6B and 6C) and suppress ETS-1 induction (Fig 6B and 6D). In contrast, constructs expressing only the first 25 or 50 residues of VSV M failed to promote hSpt16^SUMO^ degradation (Fig 6B and 6C) and did not suppress ETS-1 induction (Fig 6B and 6D). These data suggest that the N-terminal 75 amino acids of VSV M are sufficient for FEAR pathway suppression.

Consequently, we next examined N-terminal deletions of VSV M. For these studies, we took advantage of the fact that the VSV M gene actually encodes for three independent proteins; M1 (full length), M2, and M3, the latter two encoding shorter N-termini. These three VSV M proteins are expressed in the same frame but start at alternative initiation sites producing three M proteins with differing N-termini [14,40] (Fig 6E). We therefore individually expressed each of these VSV M variants and observed that only full length (M1) promoted hSpt16^SUMO^ degradation (Fig 6F and 6G) and inhibited ETS-1 induction (Fig 6F and 6H). Given that M1 and M2 N-termini differ by only 32 a.a. residues, we asked whether they might differ in their ability to interact with hSpt16 proteins. As expected, VSV M1 interacted with hSpt16/hSpt16^SUMO^. However, VSV M2 failed to interact with either form of hSpt16 (Fig 6I), suggesting the first 32 residues of VSV M, are critical for hSpt16 protein interaction, hSpt16^SUMO^ depletion, and FEAR pathway suppression.

Collectively, our data suggest that VSV M residues 1–75 are sufficient for promoting hSpt16^SUMO^ degradation indicating a critical role for the N-terminus of VSV M in FEAR pathway antagonism. Moreover, our finding that the VSV M51 residue is required for hSpt16^SUMO^ degradation, but not for hSpt16 protein binding, indicates that hSpt16 binding on its own is not sufficient to promote hSpt16^SUMO^ degradation, and that other regions of the N-terminus are involved in hSpt16 binding. Consistent with this, the M2 protein, which is missing residues 1–32 (but still retains M51), could neither bind hSpt16 proteins or promote hSpt16^SUMO^ degradation. These data suggest that hSpt16 binding, as well as an additional function mediated by the M51 residue, are both required for hSpt16^SUMO^ degradation.

### VSV M encodes an N-terminal hSpt16-binding motif important for FEAR pathway suppression

After mapping the hSpt16^SUMO^ degradation activity to the first 75 N-terminal residues of VSV M, we next examined whether structural information could provide insight into this region. Classically, full length VSV M is described to be composed of a flexible N-terminal domain (residues 1–57) known as the N-Terminal Extension (NTE) followed by a globular C-terminal domain (S8A Fig) [36,41,42]. Although a full VSV M structure for the Indiana serotype has not been solved, the crystal structure of a VSV M fragment encompassing the globular domain (a.a. 44–229) bound to Rae1-Nup98 complexes has been reported (4OWR) [42] (S8B Fig). In this structure, M contacts Rae1-Nup98 complexes using three distinct regions that project from a “fist-like” globular domain: a finger (a.a. 49–61), a thumb (a.a. 213–223), and a “web” region (a.a. 142–144) (S8B Fig). Importantly, the finger motif, encompassing the M51 residue, makes critical contacts with residues in a Rae1 β-propeller [42], illustrating the importance of this region to host interactions. Unfortunately, this structure does not contain the additional N-terminal residues (a.a. 1–43) which we have shown are critical for hSpt16/hSpt16^SUMO^ protein binding (Fig 6I), hSpt16^SUMO^ degradation (Fig 6F and 6G), and suppression of ETS-1 expression (Fig 6F and 6H). Therefore, we utilized Alphafold3 [43] to ascertain the predicted structure of full length VSV M that would include the entire N-terminus of M1 (Fig 6J). We compared this predicted structure with the 4OWR crystal structure by overlaying them (S8C Fig) and conducting pairwise similarity tests using the Research Collaboratory for Structural Bioinformatics Protein Data Bank (RSCB PDB) [44] to assign root mean square deviation (RMSD) values and template modeling (TM) scores. RMSD scores of <3 Å and TM scores >0.5 were considered to have similar overall folds while those producing RMSD values >3 Å and TM scores <0.5 were considered to be structurally unrelated [45]. Pairwise comparisons between 4OWR and our Alphafold3-predicted VSV M structure produced a RMSD of 1.46 Å and a TM score of 0.95, providing confidence in our Alphafold3-generated VSV M structure (S8D Fig). Interestingly, the AlphaFold3-generated structure predicted a small helical motif encompassing residues S3-K6 (Fig 6J). Since residues K5-L8 are conserved between the Indiana and New-Jersey serotypes [36], which both promote hSpt16^SUMO^ degradation (S6B and S6C Fig), we substituted this “KKIL” motif with alanine to determine if this motif was necessary for hSpt16^SUMO^ depletion and ETS-1 inhibition. Transient expression of VSV M-Flag encoding alanine in place of the KKIL motif failed to promote hSpt16^SUMO^ degradation (Fig 6K and 6L) or block ETS-1 expression (Fig 6K and 6M). This mutant also failed to interact with hSpt16 proteins (Fig 6N), indicating a role for this motif in VSV M-hSpt16 binding. Collectively, these data suggest that these N-terminal VSV M residues are necessary for VSV M-hSpt16 interaction.

We next asked whether M proteins from other vesiculoviruses that encode the N-terminal “KKIL” motif were capable of antagonizing the FEAR pathway. This included M proteins from Maraba virus (MARAV), Cocal virus (COCV), and Alagoas virus (VSAV) M proteins that share an overall amino acid identity with VSV M-IN of ~75–80% (S9A). To determine whether these proteins target the FEAR pathway, we transiently expressed Flag-tagged versions of these M proteins in human cells and assessed their ability to deplete hSpt16^SUMO^ and suppress ΔA51R-induced ETS-1 expression. Similar to VSV M, MARAV, COCV, and VSAV M proteins all promoted hSpt16^SUMO^ depletion (S9B and S9C Fig) and significantly reduced ETS-1 induction by ΔA51R infection (S9B and S9D Fig). Together, these data indicate that other closely-related rhabdoviruses encode M proteins that can also suppress the FEAR pathway.

### FACT contributes to VSV host range restriction in Lymantria dispar insect cells

We originally became interested in understanding poxvirus A51R protein function after discovering that these proteins rescue the post-entry abortive infection of VSV in *Lymantria dispar* (gypsy moth or spongy moth)-derived LD652 cells [12]. With the knowledge that VV A51R binds to hSpt16^SUMO^ to antagonize its antiviral function [9] and that Spt16 proteins are well-conserved across invertebrate and vertebrate eukaryotes [9], we wondered if VV A51R-mediated rescue of VSV in LD652 cells related to its ability to antagonize insect Spt16 function.

**Fig 7:**
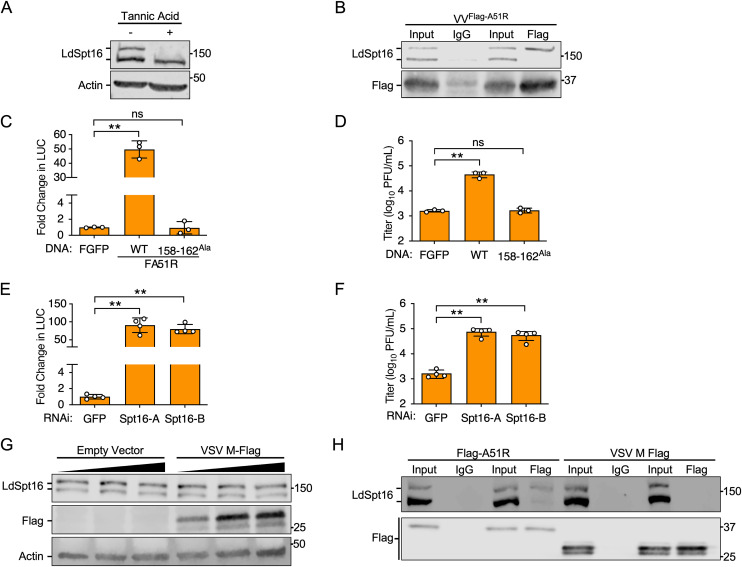
Abortive VSV replication in *L. dispar* cells can be complemented by VV A51R expression or by Spt16 RNAi. (A) IB of *L. dispar* Spt16 (LdSpt16) in LD652 whole cell extract (WCE) in the absence or presence of tannic acid (TA) treatment. (B) Co-IP of endogenous LdSpt16 with FA51R in ΔA51R^FA51R^-infected (MOI = 25) LD652 WCE. (C) Fold-change in luciferase (LUC) signal in LD652 cells transfected with indicated FA51R expression constructs for 24 h and then infected with VSV-LUC (MOI = 10) for 48 h. Fold-change in LUC is plotted as relative to LUC signal in Flag-GFP (FGFP) transfection treatments (negative control). Data are means ± SD; n = 3. (D) VSV-LUC titers from supernatants collected from C. Data are means ± SD; n = 3. (E) Fold-change in LUC signal after 72 h of transfection with dsRNAs (for RNAi) targeting either GFP (control) or LdSpt16 (Spt16-A/B) and infection with VSV-LUC (MOI = 10) for 48 h. Data are means ± SD; n = 4. (F) VSV-LUC titers from supernatants collected from E. Data are means ± SD; n = 4. (G) IB of LdSpt16 levels in LD652 WCE 48 h post-transfection with empty vector (EV) or increasing amounts of VSV M-Flag p166 expression vector. (H) Co-IP of endogenous LdSpt16 with FA51R or VSV M-Flag overexpressed in LD652 WCE. In C-F, statistical significance was determined by unpaired two-tailed Student’s t-test between indicated treatments. ** = P < 0.01, ns = not significant.

After sequencing the *L. dispar* genome [46], we discovered that hSpt16 and *L. dispar* Spt16 (LdSpt16) proteins share an overall a.a. identity of ~60% [9]. However, because VV A51R only binds SUMOylated forms of hSpt16 [9], it was important to determine if LdSpt16 was also SUMOylated. Interestingly, IBs of LD652 cell lysates revealed two LdSpt16 forms as found with hSpt16 in human cells. Treatment of LD652 cultures with tannic acid, a global SUMOylation inhibitor [47], eliminated the upper LdSpt16 form on IBs, suggesting this band represents SUMOylated LdSpt16 (LdSpt16^SUMO^) (Fig 7A). To determine if VV A51R interacts with LdSpt16^SUMO^, we infected LD652 cells with a recombinant VV strain encoding Flag-tagged A51R (FA51R) under its natural *A51R* gene promoter (ΔA51R^FA51R^) and then performed Co-IP [9]. LdSpt16^SUMO^, but not SUMOless LdSpt16 proteins, specifically co-immunoprecipitated with FA51R in LD652 cell lysates (Fig 7B), suggesting VV A51R can indeed interact with SUMOylated LdSpt16 proteins. Next, we tested if A51R-LdSpt16^SUMO^ interactions were required for A51R-mediated rescue of VSV replication in LD652 cells. To do this, we transfected LD652 cells with a control expression vector encoding Flag-tagged GFP (FGFP) or vectors encoding wild-type FA51R, or a FA51R mutant that cannot bind to SUMOylated Spt16 proteins FA51R^158-162Ala^ [9]. 48 h post-transfection, we challenged cells with a recombinant VSV strain encoding firefly luciferase (VSV-LUC) [48]. We have previously used this strain and luciferase assays to sensitively and quantitatively measure VSV gene expression in these highly restrictive insect cells [12,49]. We also collected supernatants from these LD652 cultures to assess VSV replication by plaque assay [12]. While the wild-type FA51R construct was able to significantly enhance both VSV gene expression (Fig 7C) and replication (Fig 7D), the FA51R^158-162Ala^ mutant, was completely defective in this regard. These results suggest that VV A51R-mediated rescue of VSV requires A51R-LdSpt16^SUMO^ interaction.

To directly test if LdSpt16 was involved in VSV restriction, we used *in vitro*-transcribed dsRNAs to target LdSpt16 by RNAi [12]. Compared to control RNAi targeting an irrelevant GFP mRNA sequence, VSV-LUC gene expression (Fig 7E) and replication (Fig 7F) were significantly enhanced in LD652 cell cultures transfected with two independent dsRNAs targeting LdSpt16 mRNA. These results suggested that the abortive infection of wild-type VSV in LD652 cells is at least in part dependent upon LdSpt16^SUMO^-mediated restriction. Therefore, we hypothesized that wild-type VSV M may be defective in its ability to target LdSpt16^SUMO^ for degradation. Consistent with this, VSV M-Flag transfection into LD652 cells failed to promote LdSpt16^SUMO^ depletion at any expression level tested (Fig 7G). Furthermore, VSV M-Flag failed to Co-IP with LdSpt16 proteins, in contrast to Flag-A51R proteins that we included as a positive control (Fig 7H). These results suggest that VSV M can neither bind or promote degradation of LdSpt16^SUMO^ proteins. Thus, VSV host range restriction in LD652 cells may be due to an inability of VSV M to antagonize LdSpt16^SUMO^ antiviral function.

## Discussion

**Fig 8:**
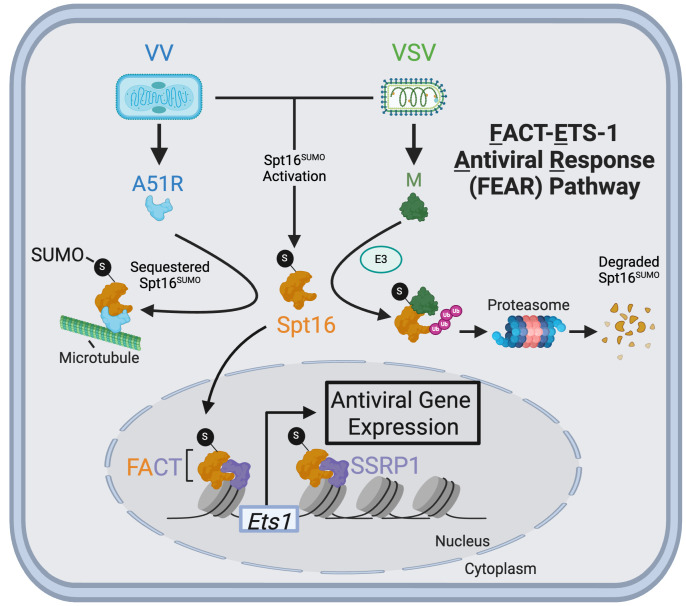
Model of FACT-ETS-1-Antiviral Response (FEAR) Pathway and its antagonism by viral pathogens. The model depicts three distinct outcomes after viral infection regarding FEAR pathway activation or evasion based on our prior published work and the data presented in this manuscript [9]. These outcomes include: 1) antagonism of the FEAR pathway by poxvirus A51R protein-mediated sequestration of Spt16^SUMO^ onto microtubules which prevents hSpt16^SUMO^ binding to SSRP1 (left) [9]; 2) antagonism of the FEAR pathway by VSV M protein-mediated degradation of Spt16^SUMO^ through the recruitment of host E3 ubiquitin ligases that specifically ubiquitinate hSpt16^SUMO^, targeting it for proteasomal degradation (right); and 3) activation of the FEAR pathway by virus infection (center), representing conditions where viral FEAR antagonists are either insufficient or non-functional (e.g., mutants). In this case, Spt16^SUMO^ translocates to the nucleus to form active FACT complexes that coordinate ETS-1 induction, resulting in an ETS-1-dependent antiviral gene expression program. Figure created with BioRender.com. Created in BioRender. Gammon, D. (2026) https://BioRender.com/fpweb0n.

Although we have previously discovered that DNA viruses activate and antagonize the FEAR pathway [9], it was less clear if this pathway was relevant to RNA virus infection. Here, we used VSV as a model RNA virus to examine if these viruses actively induce or evade the FEAR pathway. We chose this virus in part because we previously observed a measurable increase in VSV replication after hSpt16 depletion in human cells [9], hinting at the possibility that this virus may be restricted by the FEAR pathway. Furthermore, our discovery here that hSpt16 or ETS-1 depletion more strongly enhanced the replication of VSV^M51R^ versus wild-type VSV implicated VSV M in FEAR pathway suppression. However, unlike VV A51R which tethers hSpt16^SUMO^ to microtubules [9], we found VSV M interacts with hSpt16 proteins to specifically promote the proteasomal degradation of hSpt16^SUMO^ (Fig 8).

The repurposing of host E3 ubiquitin ligases to degrade antiviral host factors has been widely observed among DNA and RNA viruses [50,51]. These findings combined with our own data lead us to propose a model wherein VSV M binds with hSpt16 proteins and recruits host E3 ubiquitin ligases to promote the ubiquitination and degradation of hSpt16^SUMO^ (Fig 8). In this way, VSV M may function as an adaptor that redirects host E3 ubiquitin ligases to ubiquitinate VSV M-bound hSpt16^SUMO^. Analysis of the three VSV M forms (M1, M2, and M3) revealed the requirement of N-terminal residues to mediate hSpt16 interaction and promote hSpt16^SUMO^ degradation. Consistent with this, the KKIL motif required for hSpt16 binding is only present in M1. Additionally, conservation of this motif between IN and NJ strains suggests VSV M-hSpt16 interactions represent an important immune evasion mechanism of VSV. Given that the VSV M^M51R^ mutant was still able to bind hSpt16 proteins, but unable to promote hSpt16 protein ubiquitination or hSpt16^SUMO^ degradation, suggests that this residue may be involved in recruitment of E3 ubiquitin ligases rather than hSpt16 protein binding. Although the VSV M^M51R^ mutant has been previously shown to be unable to interact with the Rae1-Nup98 complex [17], our data demonstrate that Rae1 or Nup98 do not influence VSV-mediated hSpt16^SUMO^ depletion. Thus, because the M51 residue resides in a “finger-like” projection that protrudes from the globular domain of VSV M [42], it may be a docking site for not only Rae1 but also for additional host factors, such as E3 ubiquitin ligases.

Given that VSV only targets hSpt16^SUMO^, and not SUMOless hSpt16, for degradation, it is possible that VSV M recruits a SUMO-Targeted Ubiquitin Ligase (STUbL). STUbLs are E3 ubiquitin ligases which only recognize SUMOylated proteins as ubiquitination substrates [52,53]. STUbLs typically recognize mono- or poly-SUMOylated proteins and conjugate ubiquitin onto lysines on the target protein directly, or the attached SUMO moiety, to promote their ubiquitination [52]. Ubiquitination by STUbLs can result in changes in target protein turnover, localization, or function [52,54]. Thus, potential recruitment of these specialized SUMO-targeted E3 ubiquitin ligases may explain why VSV M binds both hSpt16 and hSpt16^SUMO^, but only promotes degradation of the latter. The identification of E3 ubiquitin ligases potentially involved in VSV M-mediated hSpt16^SUMO^ degradation is an active area of investigation.

We observed no difference in ETS-1 induction during wild-type or VSV M^M51R^ infection in cells deficient in the key IFN response mediators IRF3 and STAT1. This finding is consistent with our prior work showing that DNA viruses activate the FEAR pathway independently of the IFN response [9]. Thus, the FEAR pathway can be included among other IFN-independent mechanisms that host cells use to restrict RNA virus replication [55–57]. The IFN-independent nature of the FEAR pathway is consistent with the evolutionary conservation of Spt16 and ETS-1 in invertebrates (which lack IFNs) and suggests that the FEAR pathway is an ancient antiviral program predating the emergence of IFN-based immunity.

This study has also provided a key insight into the poorly understood abortive infection of VSV in lepidopteran insect cells and the rescue of this virus in these cells by poxvirus A51R proteins [12]. We previously found Spt16 proteins from various mammalian and invertebrate species cell lysates to exhibit the same, two-band pattern on IBs found in human cell extracts, suggesting SUMOylation of Spt16 proteins is highly conserved among both vertebrate and invertebrate eukaryotes [9]. More formal evidence for this was provided here where we demonstrated that treatment with a SUMOylation inhibitor (tannic acid) [47] could specifically eliminate the higher molecular weight form of LdSpt16 in *L. dispar*-derived LD652 cells. Strikingly, despite being encoded by a mammalian poxvirus, VV A51R was able to interact with LdSpt16^SUMO^. This is likely due to the high degree of conservation between human and Spt16 proteins [9]. Our findings that: 1) VSV M cannot bind nor deplete LdSpt16^SUMO^ levels; 2) A51R proteins lacking a functional Spt16-binding domain cannot rescue VSV replication in *L. dispar* cells; and 3) LdSpt16 RNAi rescues VSV replication, all strongly implicate LdSpt16^SUMO^ in VSV host range restriction in these cells. Importantly, these results also suggest an ancient role for SUMOylated Spt16 proteins in eukaryotic antiviral immunity. Future studies will be needed to determine if insect ETS transcription factors [58] are also involved in virus restriction and whether the FEAR pathway is indeed intact in invertebrates.

Understanding FACT and ETS-1 regulation and function is important not only because of their relevance to antiviral immunity, but because their dysregulation in human malignancies is strongly associated with poor cancer prognosis [59,60]. FACT upregulation correlates with aggressive tumor phenotypes and has motivated the development of FACT inhibitor drugs, which are currently in clinical trials [59,61]. ETS-1 is a proto-oncoprotein and its dysregulation can promote cellular transformation and invasion [62,63]. Thus, the FEAR pathway engages host factors that are both antiviral and pro-tumorigenic, raising the possibility that it may influence oncolytic virus efficacy in transformed cells. Interestingly, VSV M^∆M51/M51R^ strains are being pursued as oncolytic agents and are currently in phase I clinical trials [64,65]. However, some cancer cell types have been shown to be refractory to infection by these mutant VSV strains, limiting their use in oncolytic virotherapy [35,66]. It is possible that FEAR pathway activation in these refractory cancer cell types may contribute to the restriction of these oncolytic VSV^∆M51/M51R^ strains. Accordingly, combining these oncolytic VSV^∆M51/M51R^ strains with FACT inhibitor drugs [67] may represent a rational and unexplored strategy to improve therapeutic outcomes when targeting cancers normally refractory to these oncolytic agents.

In conclusion, our work has demonstrated that although RNA viruses can activate the FEAR pathway, they also encode mechanisms to counter this response. Thus, it is clear that the FEAR pathway is relevant to RNA virus host interactions. Major questions remain regarding how RNA viruses activate the FEAR pathway and how ETS-1 expression eventually leads to virus restriction. Studies are ongoing to explore these exciting areas of FEAR pathway biology. Moreover, whether other RNA virus families antagonize the FEAR pathway is an active area of investigation. Ultimately, a greater understanding of the activation and evasion of the FEAR pathway by viruses will help us better appreciate the role of this host response in the evolutionary arms race between viruses and their hosts.

## Materials and Methods

### Cell lines and primary cultures

Mammalian cell lines were maintained at 37°C in 5% CO_2_ atmosphere. A549, U2OS, 293T, and BHK-21 cells were cultured in DMEM supplemented with 10% FBS. BSC-40 cells were passaged in MEM containing 5% FBS. All media above additionally contained 1% non-essential amino acids, 1% L-glutamine, and 1% antibiotic/antimycotic. NHDF cells were passaged in fibroblast basal medium supplemented with low serum fibroblast growth kit. LD652 were cultured in a 1:1 mixture of Ex-Cell 420 and Graces insect medium supplemented with 10% FBS at 27°C under normal atmospheric conditions [12,68].

### Viruses and virus titrations

All VSV strains were amplified using low MOI infections in BHK-21 cells. BSC-40 cells were used for VV stock preparation [12,69]. Unless otherwise noted, virus strains were titrated by plaque assay (VSV-LUC, ΔA51R^FA51R^, ΔA51R) or fluorescent foci/plaque assay (VSV-eGFP, VSV^M51R^-eGFP, VSV^∆M51^-GFP) on A549, U2OS, or BSC-40 cell monolayers, with a 1.5% low-melting point agarose (Invitrogen) overlay used for VSV titrations [9]. Experimental viral infections were incubated for 1 h in serum free DMEM at 37°C before the inoculum was replaced with complete media for the remainder of the infection. Where indicated, 25 nM bortezomib was added 6 h post VSV-eGFP infection and was kept in the media until the end of the experiment. At indicated times post-infection, infected cell culture supernatants were collected, clarified by centrifugation, and clarified supernatant titers were determined by serial dilution followed by plaque assay or fluorescent foci/plaque assay [9].

### Expression vectors

The human ETS-1-HA pEZ-M07 and the control expression vector were obtained from GeneCopoeia. VSV P-Flag, VSV N-Flag, VSV M(NJ)-Flag, VSV M(IN)-Flag, MARAV M-Flag, COCV M-Flag, VSAV M-Flag, and M51R/ΔM51 mutant derivatives were constructed by gene synthesis (Gene Universal or TWIST BioScience) with inclusion of 5’-*Sac*II and 3’-*Pac*I sites for cloning into *Sac*II/*Pac*I-digested pcDNA3 [9]. VSV M2, M3, and KKIL^Ala^ variants were constructed by PCR amplification with inclusion of *Sac*II and *Pac*I sites for cloning into *Sac*II/*Pac*I-digested pcDNA3 [9]. VSV M-Flag was also cloned into the insect p166 expression vector [12] using *Sac*II/*Pac*I sites. A.a. 1–229, -150, -100, -75, -50, and -25 VSV M-N-terminal GFP-Flag constructs were PCR amplified with the inclusion of 5’-*BamHI* and 3’-*SacII* sites for cloning into BamHI/SacII-digested GFP-Flag pcDNA3 [9]. His, VSV M-His or M^M51R^-His was cloned into a pGEX vector containing a N-terminal GST using 5’-*BamHI* and 3’-*SalI* sites. VSV G-Flag was PCR amplified with inclusion of *Sac*II and *Pac*I sites for cloning into *Sac*II/*Pac*I-digested pcDNA3. Wild-type VV Flag-A51R and Flag-A51R^158-162Ala^ open reading frames were cloned into the *Sac*II/*Pac*I sites of p166 by *Sac*II/*Pac*I digest of Flag-A51R and Flag-A51R^158-162Ala^ pcDNA3 vectors [9]. The HA-hSpt16 and HA-hSpt16^∆NLS^ pEZ-M06 expression vectors have been described [9]. The Flag-GFP pcDNA3 and p166 vectors have been described [9,12]. All genes were sequence verified. Expression plasmids were transfected into U2OS cell lines as previously described using Lipofectamine 2000 in Opti-MEM I [12]. Plasmids transfected into 293T cells used a 1:6 ratio of DNA:PEI. Plasmids transfected into LD652 cells used a 1:3 ratio of DNA:Cellfectin II [49]. All cells were incubated with the transfection mixture for 6 h and then media was replaced with complete culture media Cells were incubated for the indicated times prior to further processing (e.g., protein extraction, see below).

### General protein extraction and inhibitor treatments

Cells were washed with phosphate-buffered saline (PBS) prior to scraping and transfer into a 1.5 mL microcentrifuge tube for centrifugation at 4,000 x *g* at 4°C for 15 min. Cell pellets were resuspended in either 1x Reporter Lysis Buffer containing 1% Protease Inhibitor Cocktail (PIC) and 1 mM phenylmethylsulfonyl fluoride (PMSF) and freeze-thawed once, or were resuspended in Pierce RIPA buffer (containing 1% PIC and 1 mM PMSF) prior to addition of 5x SDS-PAGE loading buffer (100 mM tris HCl, pH 6.8, 4% SDS, 12% (v/v) glycerol, 4 mM DTT, 0.02% (w/v) Bromophenol Blue). Where indicated, cells were treated with 10 µM tannic acid for 4 h prior to protein harvest. Also, where indicated, TAK-243 (1 μM), bortezomib (25 nM), MG-132 (40 μM), or DMSO (vehicle control) was added to cells 6 h post-transfection and was kept in media until protein extraction.

### Immunoblotting

WCE were boiled for 10 min prior to SDS-PAGE electrophoresis at 65–100 V for approximately 2–4 h. Separated proteins were transferred in Towbin Buffer onto nitrocellulose membranes at 150 mA at 4°C for 90 min and blocked with Li-COR Odyssey Blocking Buffer for 1 h at RT. Membranes were blotted with primary Ab overnight at 4°C, with actin serving as a loading control. After 3 x 5 min PBS-T (PBS, 0.1% Tween) washes, membranes were incubated in secondary Ab conjugated to an IRDye for 1 h, washed 3 x 5 min in PBS-T, then a final 5 min PBS wash. Membranes were then imaged with an Li-COR Odyssey Fc Imager. Densitometric analysis of immunoblots were performed using ImageJ (http://imagej.nih.gov/ij, Java 1.8.0_172[54-bit]). Digital images of immunoblots were analyzed by measuring the integrated density of each protein band following local background subtraction. The intensity of each target band (e.g., ETS-1, hSpt16^SUMO^, hSpt16 or SSRP1) was normalized to the corresponding loading control band (e.g., actin) within the same lane from the same membrane. Normalized values were calculated as the ratio of target protein signal to actin signal and were subsequently expressed and plotted as “fold-change” relative to the indicated experimental control sample. The quantification shown represents the mean normalized values from at least three independent biological replicates with representative immunoblots shown in figures corresponding to one of these independent experiments.

### Immunoprecipitation

Transient mammalian co-immunoprecipitations were performed by seeding 12e6 293T cells overnight, followed by PEI-mediated transfection for 6 h when bortezomib containing complete media was replaced. Infection-based co-immunoprecipitations were performed by seeding 15e6 A549 cells overnight, then infected for 24 h in the presence of bortezomib added after 6 hpi. Infection-based LD652 co-immunoprecipitation experiments were performed by seeding 1 x 10^6^ LD652 overnight, then infected with ∆A51R^FA51R^ (MOI = 25) for 24 h in the presence of AraC (0.2mg/ml) added with the replacement media. Regardless of co-immunoprecipitation condition, cells were washed with equal volumes of PBS twice prior to cell lysis in IP lysis buffer (1% PIC, 1 mM PMSF, 25 mM Tris-HCl at pH 7.4, 150 mM NaCl, 1.0% NP-40) and subjected to shearing and sonication (two-15 second sonications with a 30 second interval on ice). Samples were benzonase treated (250 units/mL) for 1 h at RT. 10% of “input” was collected, and remaining lysate was end-over-end incubated with 10 µg of primary Ab overnight at 4°C (rabbit-anti-Flag, mouse-anti-VSV M or isotype control Ab). Then, lysates were incubated with PureProteome protein A/G magnetic beads for 1–2 h, extensively washed, and immunoprecipitants eluted in 60 μl 2x SDS-PAGE loading buffer. IPs of total SUMOylated protein fractions in mock-, VSV-eGFP, or VSV^M51R^-eGFP-infected (MOI = 10) A549 cells for 12 h used either SUMO-1 or SUMO-2/3 Ab included with the Cytoskeleton Signal Seeker SUMOylation 1 or 2/3 Detection Kit and IPs were carried out according to the manufacturer instructions [9]. TUBE pulldowns were performed as per the manufacturer instructions. Briefly, cells were lysed in cell lysis buffer (50 mM Tris-HCl, pH 7.5, 150 mM NaCl, 1 mM EDTA, 1% NP-40, 10% glycerol, 50 µM PR-619, 1% PIC, 1 mM PMSF) and incubated with TUBE2 or control magnetic beads at 4 °C overnight, followed by extensive washing with TBS-T (20 mM Tris-HCl, pH 8.0, 0.15 M NaCl, 0.1% Tween-20).

### Protein purification

GST-His only, GST-VSV M-His, and GST-VSV M^M51R^-His plasmids were transformed in BL21-CodonPlus(DE3)-RIPL cells in the presence of 100 μg/ml ampicillin. Once overnight cultures reached an OD_600_ of 0.5, protein expression was induced with 0.5mM isopropyl-D-1-thiogalactopyranoside (IPTG) at 18˚C for 20 h. Cells were lysed using an Emulsiflex C5 in GST Buffer A (50 mM Tris-HCl, 500 mM NaCl, 1 mM DTT, 1% PIC, 1 mM PMSF, 1 μM Pepstatin, 2 μM Aprotinin, 10 μM Leupeptin, 0.2 mM AEBSF, and 10% glycerol, pH 7.5). Proteins were affinity purified using Glutathione Superflow Agarose resin and washed in GST Buffer A prior to elution using GST Buffer B (50 mM Tris-HCl, 500 mM NaCl, 1 mM DTT, 1% PIC, 1 mM PMSF, 10% glycerol, and 10 mM glutathione, pH 7.5). The protein was then passed through a HisTrap HP column, subsequently washed with 10 volumes of His Buffer A (50 mM Tris-HCl, 150 mM NaCl, 1 mM DTT, 1 mM PMSF, 50 mM imidazole, 10% glycerol, pH 7.5) then eluted in His Buffer B (50 mM Tris-HCl, 150 mM NaCl, 1 mM DTT, 1 mM PMSF, 500 mM imidazole, 10% glycerol, pH7.5). Proteins were concentrated in a 10 kDa MWCO ultra centrifugal filter prior to storage. Flag-hSpt16 proteins were purified as previously described [9].

### In vitro pulldown

10 μg of purified GST-His, GST-VSV M-His or GST-VSV M^M51R^-His proteins were co-incubated with 10 μg of purified Flag-Spt16 proteins in Pull Down Buffer (0.1% NP40, 100 mM NaCl, 50 mM Tris-HCl pH 7.4 with 1% PIC and 1 mM PMSF) overnight. The next day, 10% input aliquots were taken and the rest of the sample was incubated with glutathione agarose resin for 1 h, washed 5 times in Pull Down Buffer, then eluted in 2 x SDS-PAGE loading dye for SDS-PAGE analysis.

### RNAi in cell culture

All siRNAs were obtained from Sigma’s pre-designed siRNA library (see S1 Table). The hSpt16 and ETS-1-targeted siRNAs have been previously validated [9]. Transient siRNA-mediated knockdown was achieved by reverse transfecting mammalian cells with Lipofectamine 2000 according to manufacturer’s protocol for 48–72 h prior to viral infection [9]. RNAi knockdowns in LD652 cells were performed as previously described using *in vitro*-transcribed dsRNAs transfected with Cellfectin II in Sf-900 II serum free medium [12]. Transfection medium was replaced with complete culture medium after overnight culture. Sequences of primers used to generate dsRNA targeting GFP (control) or *LdSpt16* gene sequences are in S1 Table.

### Luciferase assays

Luciferase assays in LD652 WCE were performed as previously described with minor modifications [12,49]. Briefly, at the indicated times post-infection, cells were washed in PBS, collected by centrifugation (2000 rpm, 10 min, 4°C) and lysed in reporter lysis buffer (Promega). Lysates were spotted to 96-well dishes, mixed with Luciferase Assay Reagent (Promega) and arbitrary light units (LU) were measured using an FLUOstar Omega Plate reader (BMG Labtech).

### Antibodies

Information regarding antibodies used in this study, including their source, is available in S1 Table.

### Fluorescence microscopy

At indicated times post-infection, cells were stained with serum free media containing NucBlue (Thermo Fisher) for 30 min followed by replacement with equal volumes of PBS. Cells were imaged using a 4X objective on an EVOS-FL fluorescence microscope (Thermo Fisher) using DAPI and GFP cubes. Each condition had three replicate wells, and at least four images/well were collected for analysis. Image analysis was conducted using Fiji (NIH) to quantify the normalized GFP signal/field by dividing total GFP signal by total DAPI (NucBlue) signals. Positive signal was determined by using uninfected wells lacking NucBlue stain to set a minimum threshold in both DAPI and GFP channels that was applied to the remaining dataset. Fold change in GFP signals were calculated by dividing normalized GFP values of experimental treatments with normalized GFP values in control treatments (indicated in each Figure).

### Immunofluorescence microscopy

For staining U2OS cells transiently transfected with HA-hSpt16 or HA-hSpt16^∆NLS^, cells were seeded at a density of 30,000 cells per coverslip, cultured overnight, then transiently transfected with 500 ng of HA-hSpt16 or HA-hSpt16^∆NLS^ expression plasmids with Lipofectamine2000 for 6 h before replacing with complete media. Cells were fixed with 4% paraformaldehyde at 24 h post-transfection, incubated with blocking buffer (PBS with 1% BSA and 0.1% Triton-X) for 1 h, stained with rabbit-anti-HA Ab for 2 h, washed extensively with blocking buffer, incubated with Alexa Fluor-conjugated secondary Ab for 1h, and then again washed extensively. Coverslips were then mounted onto glass slides using ProLong™ Diamond anti-fade with DAPI and imaged with an Olympus FV10i confocal laser scanning microscope (version 2.1) equipped with Olympus Fluoview software (version 4.2a). Images were deconvoluted with CellSens Imaging software (version 1.18).

### Protein sequence alignment and structure prediction

VSV M (NCBI accession: NP_041714.1) structure was predicted using AlphaFoldv3 [43] and analyzed using PyMOL **(**The PyMOL Molecular Graphics System **version 1.2r3pre, Schrödinger, LLC.).** To determine structural similarity between VSV M 4OWR and our Alphafold3-generated VSV M structure, pairwise structural alignments were conducted using RSCB PDB [44] to assign RMSD and TM scores. RMSD values of <3 Å and TM scores >0.5 were considered to have similar overall fold, while pairwise comparisons producing RMSD values >3 Å and TM scores <0.5 were considered to be structurally unrelated [45].

### Statistical analyses

All statistical analyses were conducted using GraphPad Prism (version 8.0) software and *P* values <0.05 were considered statistically significant. Sample sizes, statistical tests used, and *P* value information are indicated in the respective Figure or Figure legend for each quantitative experiment.

## Materials availability

All unique/stable reagents generated in this study are available from the lead contact with a completed material transfer agreement. Specific details regarding the source of all key experimental reagents (primers, plasmids, Abs, viruses, cell lines, etc.) can be found in S1 Table.

## Supporting information

S1 Fig VSV-induced ETS-1 is independent of IFN signaling. (A-B) Representative IB (A) and quantification (B) of ETS-1 expression using IB of WCE from control or IRF3 KO A549 cells infected with mock-, VSV-eGFP (WT), or VSV^M51R^-eGFP (M51R) (MOI = 10) for 8 h. (C-D) Representative IB (C) and quantification (D) of ETS-1 expression using IB of WCE from control or STAT1 KO A549 cells infected with mock-, WT, or M51R (MOI = 10) for 8 h. Data are means ± SD; n = 3. Statistical significance was determined by unpaired two-tailed Student’s t-test between indicated treatments. * = P < 0.05; ** = P < 0.01; ns, not significant.
(TIF)

S2 Fig The VSV^M51R^-eGFP strain is unable to deplete hSpt16^SUMO^, regardless of MOI. IB of endogenous hSpt16 in A549 WCE after infection with VSV-eGFP (WT) or VSV^M51R^-eGFP (M51R) at the indicated MOI. GFP is used as a marker for infection.
(TIF)

S3 Fig The FEAR pathway contributes to VSV restriction in primary human cells. (A-B) VSV-eGFP (WT) and VSV^M51R^-eGFP (M51R) titers 24 hpi (MOI = 0.001) in NHDF cells transfected with indicated RNAi treatments using two independent siRNAs for either hSpt16 (A) or ETS-1 (B) knockdown. Scram., scrambled siRNA. (C) IB of endogenous hSpt16 and ETS-1 in NHDF WCE after infection with WT or M51R (MOI = 10). (D-G) Densitometric quantification of ETS-1 (D), hSpt16^SUMO^ (E), SUMOless hSpt16 (F), and SSRP1 (G) from multiple IB experiments as in C. Data are means ± SD; n = 3. In D-G, results of unpaired two-tailed Student’s t-test comparing protein levels in mock WCE to infected WCE are shown above each bar graph as: * = P < 0.05, ** = P < 0.01, or ns = not significant.
(TIF)

S4 Fig A VSV^∆M51^ strain is unable to deplete intracellular hSpt16^SUMO^ levels. IB of endogenous hSpt16 in A549 WCE after infection with VSV-GFP (WT) or VSV^∆M51^-GFP (∆M51) (MOI = 3). GFP is used as a marker for infection.
(TIF)

S5 Fig VSV M promotes cytoplasmic hSpt16^SUMO^ depletion. (A) Immunofluorescence (IF) images of U2OS cells transfected with indicated HA-tagged hSpt16 constructs for 24 h. Scale bar = 5 μm. (B) IB of 293T WCE 24 h post-transfection with indicated HA-tagged hSpt16 [9] constructs along with either empty vector (EV), VSV M-Flag (WT), or VSV M^M51R^-Flag (M51R) pcDNA3 vectors. Asterisks indicate probable VSV M degradation products.
(TIF)

S6 Fig Both Indiana (IN) and New Jersey (NJ) encoded VSV M proteins deplete hSpt16^SUMO^ and suppress ETS-1. (A) N-Terminal sequence alignment of M proteins from VSV-Indiana (IN) or New Jersey (NJ). (B) IB of endogenous hSpt16 and ETS-1 in U2OS WCE after transient transfection of empty vector (EV) or VSV M-Flag from serotype IN or NJ infected with ∆A51R VV for ETS-1 induction (VV^∆A51R^) (MOI = 10). (C-D) Densitometric quantification of hSpt16^SUMO^ (C) or ETS-1 (D) from multiple IB experiments as in B. Data are means ± SD; n = 3. Results of unpaired two-tailed Student’s t-test between protein levels in empty vector (EV) mock WCE and infected WCE are shown above each bar graph as: ** = P < 0.01 or ns = not significant.
(TIF)

S7 Fig VSV M-mediated depletion of hSpt16^SUMO^ is independent of Rae1 and Nup98. (A-B) IB of endogenous hSpt16 in A549 WCE 72 h after RNAi of Rae1 (A) or Nup98 (B) under mock- or VSV-eGFP-infection (MOI = 10) conditions for 12 h. VSV N and GFP are markers for infection. Scram., scrambled.
(TIF)

S8 Fig VSV M protein structural modeling. (A) VSV M protein diagram aligning the first 75 residues in VSV M IN and NJ. Met1 (M1, start of M1), Met33 (M33, start of M2), and Met51 (M51, start of M3) residues are indicated in red. KKIL motif is indicated in orange. NTE = N-terminal extension. α1 = alpha helix 1 based on AlphaFold3 prediction. Protein map based on [42]. (B) 4OWR structure of VSV M fragment (a.a. 44–229) bound to Rae1-Nup98 complex 4. (C) Overlay of structures from B and AlphaFold3-predicted structure of full length VSV M IN. (D) Results of pairwise comparison of structures in C showing RMSD (in Å) and TM values.
(TIF)

S9 Fig M proteins from other vesiculoviruses are capable of FEAR pathway antagonism. (A) Overall percent amino acid (a.a.) identity of full-length vesiculovirus M proteins relative to VSV M-IN with sequence alignment of the first 30 a.a. of the indicated M proteins highlighting the “KKIL” motif in orange. (B) Representative IB of endogenous hSpt16 and ETS-1 in U2OS WCE following transient transfection with empty vector (EV) or Flag-tagged M proteins from VSV-IN, Maraba virus (MARAV), Cocal virus (COCV), and Alagoas virus (VSAV). Cells were infected with ΔA51R (VV^ΔA51R^; MOI = 10) to induce ETS-1 expression. (C–D) Densitometric quantification of hSpt16^SUMO^ (C) and ETS-1 (D) from multiple independent immunoblot experiments performed as in (B). Data are means ± SD; n = 3. Results of unpaired two-tailed Student’s t-test between protein levels in empty vector (EV) WCE and WCE expressing Flag-tagged proteins are shown above each bar graph as: ** = P < 0.01 or ns = not significant.
(TIF)

S1 Table Key resources and reagents. (XLSX)

S2 Table Quantified data. (XLSX)
