# Supplementary figures and images for: The vesicular stomatitis virus matrix protein promotes FACT subunit depletion to suppress the FEAR pathway
Source: PLoS Pathog. 2026 Jul 17;22(7):e1014430. doi: 10.1371/journal.ppat.1014430 (PMC13395346; doi:10.1371/journal.ppat.1014430)

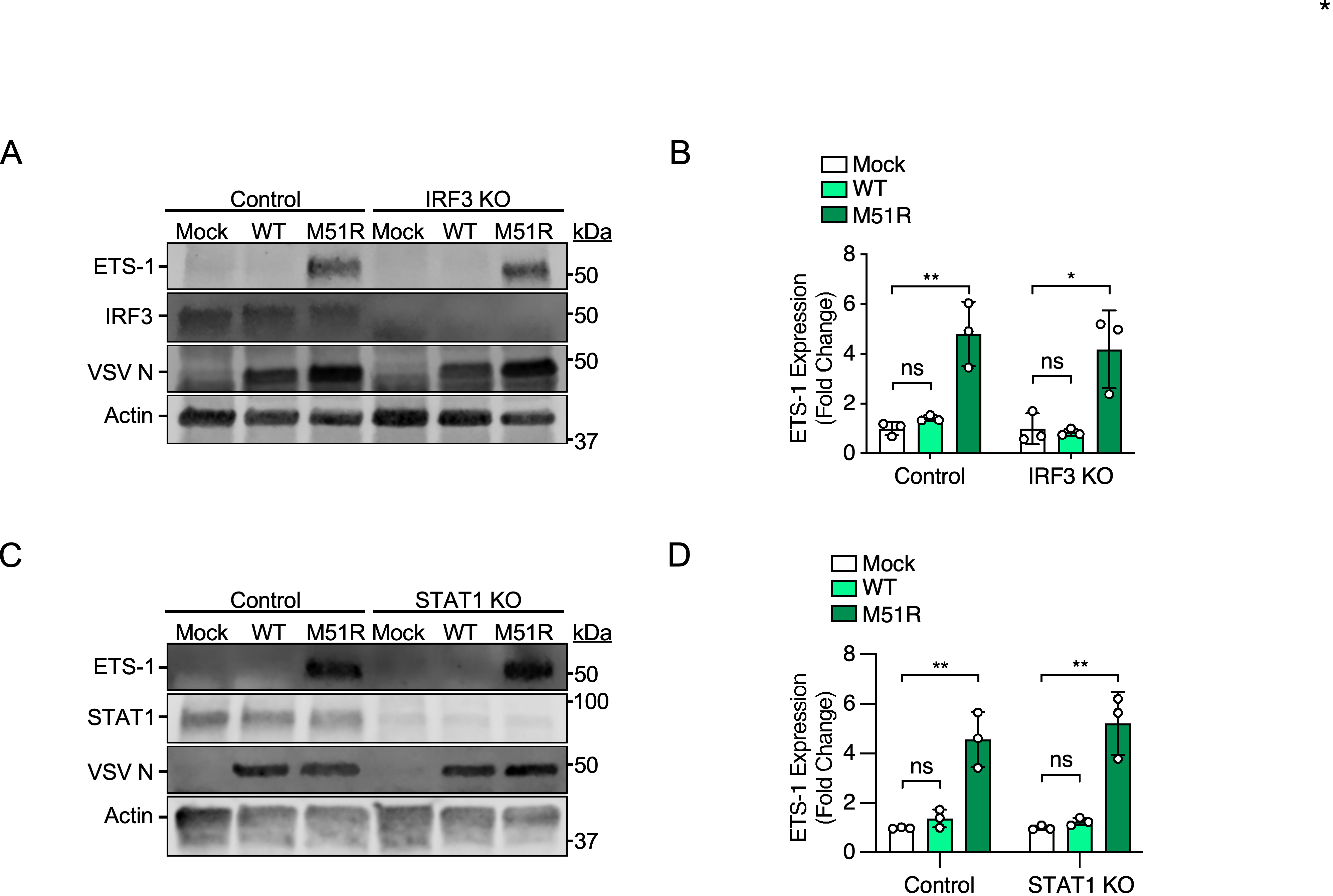

Supplement: S1 Fig — (A-B) Representative IB (A) and quantification (B) of ETS-1 expression using IB of WCE from control or IRF3 KO A549 cells infected with mock-, VSV-eGFP (WT), or VSVM51R-eGFP (M51R) (MOI = 10) for 8 h. (C-D) Representative IB (C) and quantification (D) of ETS-1 expression using IB of WCE from control or STAT1 KO A549 cells infected with mock-, WT, or M51R (MOI = 10) for 8 h. Data are means ± SD; n = 3. Statistical significance was determined by unpaired two-tailed Student’s t-test between indicated treatments. * = P < 0.05; ** = P < 0.01; ns, not significant. (TIF) [file ppat.1014430.s001.tif]

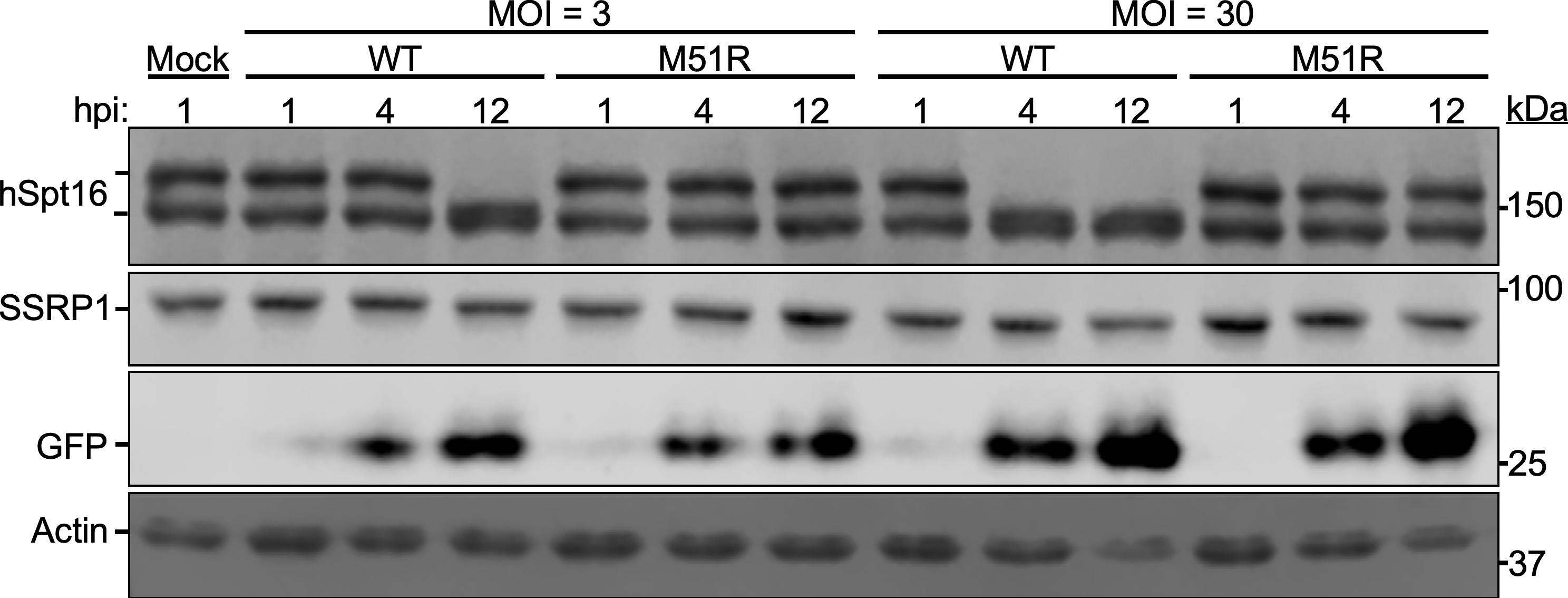

Supplement: S2 Fig — IB of endogenous hSpt16 in A549 WCE after infection with VSV-eGFP (WT) or VSVM51R-eGFP (M51R) at the indicated MOI. GFP is used as a marker for infection. (TIF) [file ppat.1014430.s002.tif]

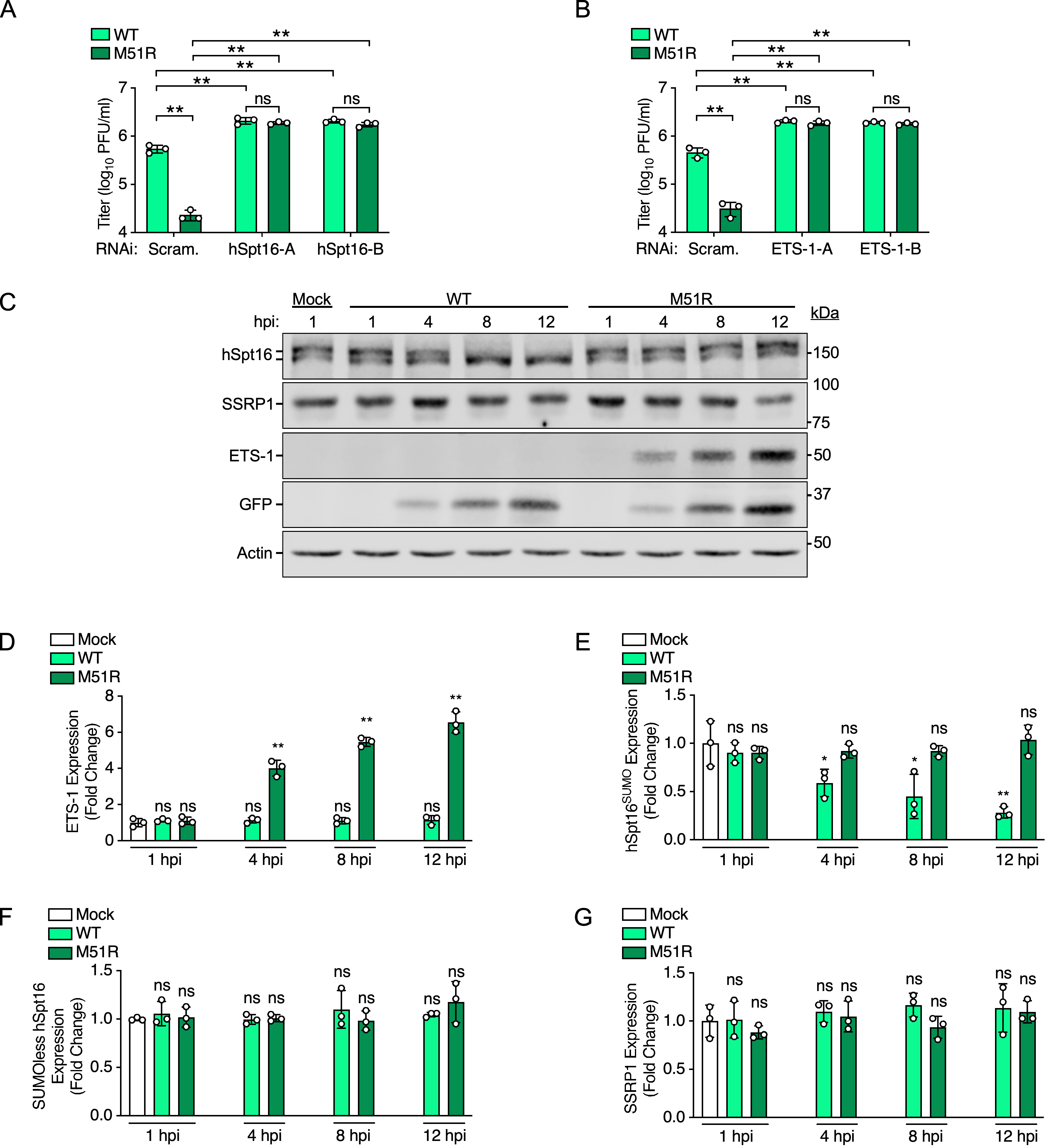

Supplement: S3 Fig — (A-B) VSV-eGFP (WT) and VSVM51R-eGFP (M51R) titers 24 hpi (MOI = 0.001) in NHDF cells transfected with indicated RNAi treatments using two independent siRNAs for either hSpt16 (A) or ETS-1 (B) knockdown. Scram., scrambled siRNA. (C) IB of endogenous hSpt16 and ETS-1 in NHDF WCE after infection with WT or M51R (MOI = 10). (D-G) Densitometric quantification of ETS-1 (D), hSpt16SUMO (E), SUMOless hSpt16 (F), and SSRP1 (G) from multiple IB experiments as in C. Data are means ± SD; n = 3. In D-G, results of unpaired two-tailed Student’s t-test comparing protein levels in mock WCE to infected WCE are shown above each bar graph as: * = P < 0.05, ** = P < 0.01, or ns = not significant. (TIF) [file ppat.1014430.s003.tif]

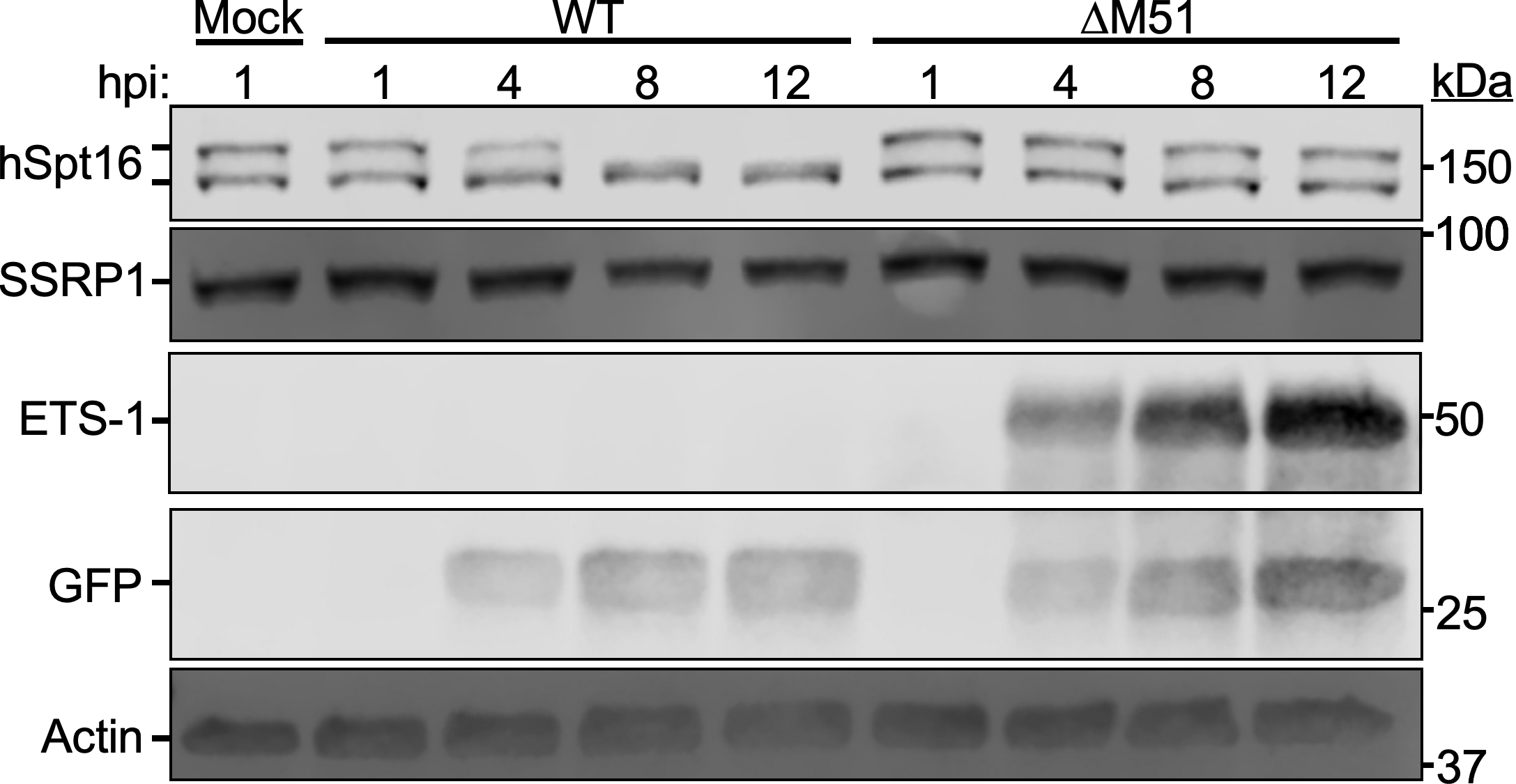

Supplement: S4 Fig — IB of endogenous hSpt16 in A549 WCE after infection with VSV-GFP (WT) or VSV∆M51-GFP (∆M51) (MOI = 3). GFP is used as a marker for infection. (TIF) [file ppat.1014430.s004.tif]

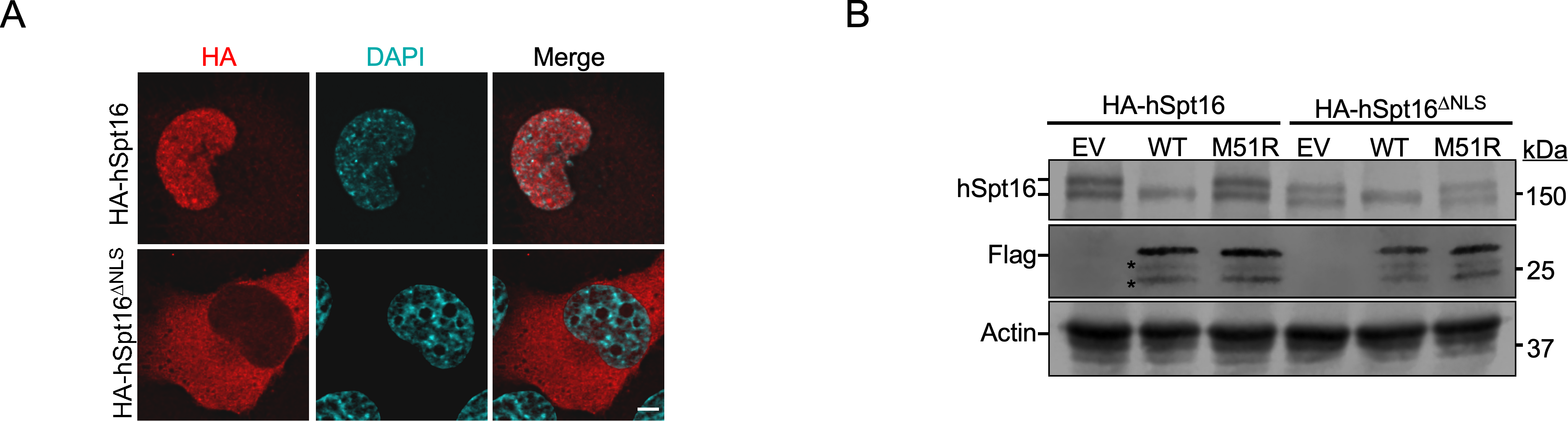

Supplement: S5 Fig — (A) Immunofluorescence (IF) images of U2OS cells transfected with indicated HA-tagged hSpt16 constructs for 24 h. Scale bar = 5 μm. (B) IB of 293T WCE 24 h post-transfection with indicated HA-tagged hSpt16 [9] constructs along with either empty vector (EV), VSV M-Flag (WT), or VSV MM51R-Flag (M51R) pcDNA3 vectors. Asterisks indicate probable VSV M degradation products. (TIF) [file ppat.1014430.s005.tif]

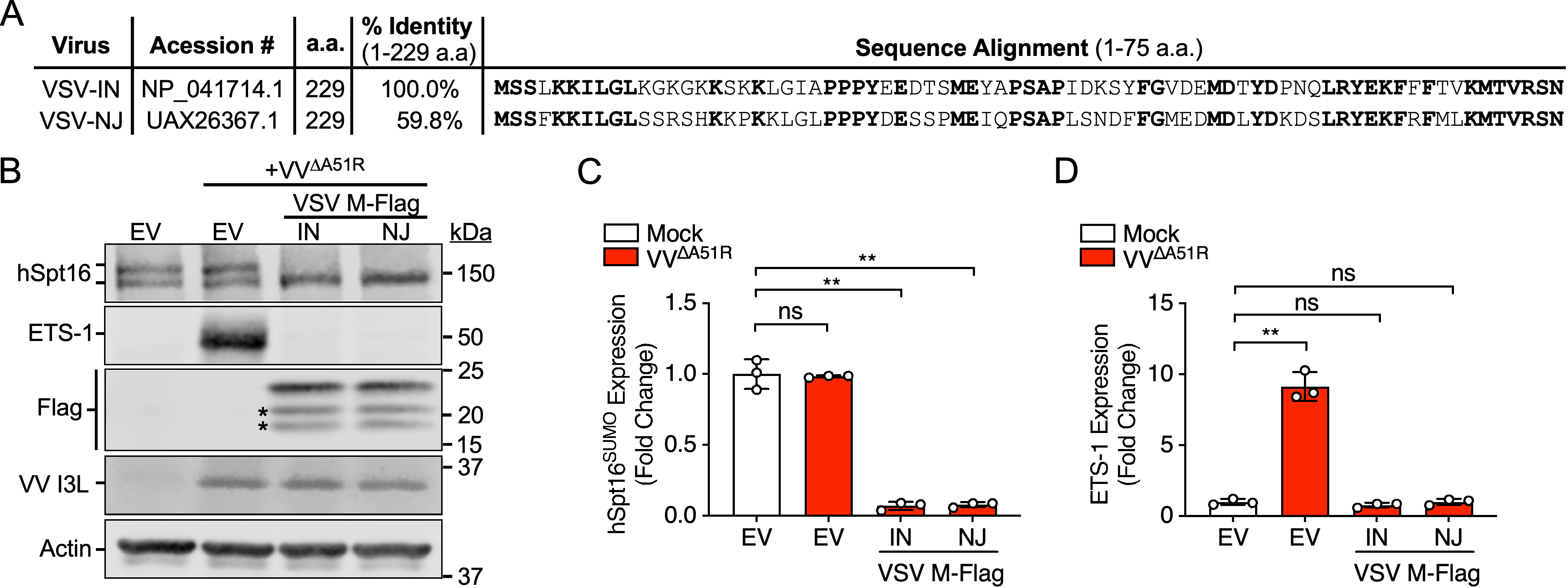

Supplement: S6 Fig — (A) N-Terminal sequence alignment of M proteins from VSV-Indiana (IN) or New Jersey (NJ). (B) IB of endogenous hSpt16 and ETS-1 in U2OS WCE after transient transfection of empty vector (EV) or VSV M-Flag from serotype IN or NJ infected with ∆A51R VV for ETS-1 induction (VV∆A51R) (MOI = 10). (C-D) Densitometric quantification of hSpt16SUMO (C) or ETS-1 (D) from multiple IB experiments as in B. Data are means ± SD; n = 3. Results of unpaired two-tailed Student’s t-test between protein levels in empty vector (EV) mock WCE and infected WCE are shown above each bar graph as: ** = P < 0.01 or ns = not significant. (TIF) [file ppat.1014430.s006.tif]

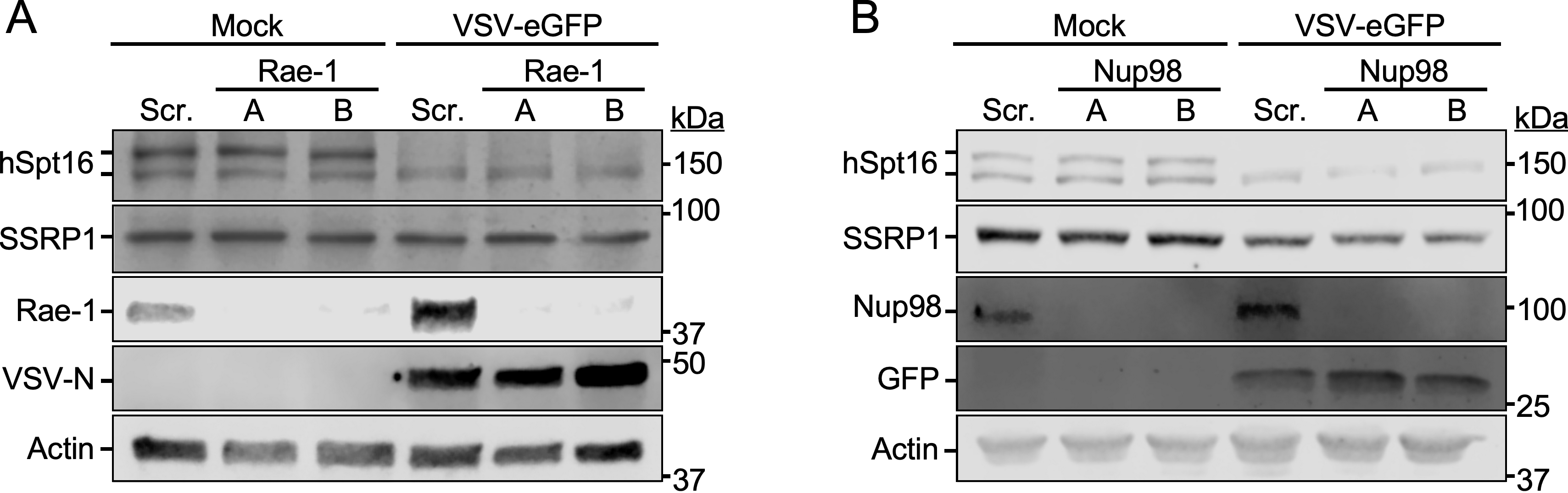

Supplement: S7 Fig — (A-B) IB of endogenous hSpt16 in A549 WCE 72 h after RNAi of Rae1 (A) or Nup98 (B) under mock- or VSV-eGFP-infection (MOI = 10) conditions for 12 h. VSV N and GFP are markers for infection. Scram., scrambled. (TIF) [file ppat.1014430.s007.tif]

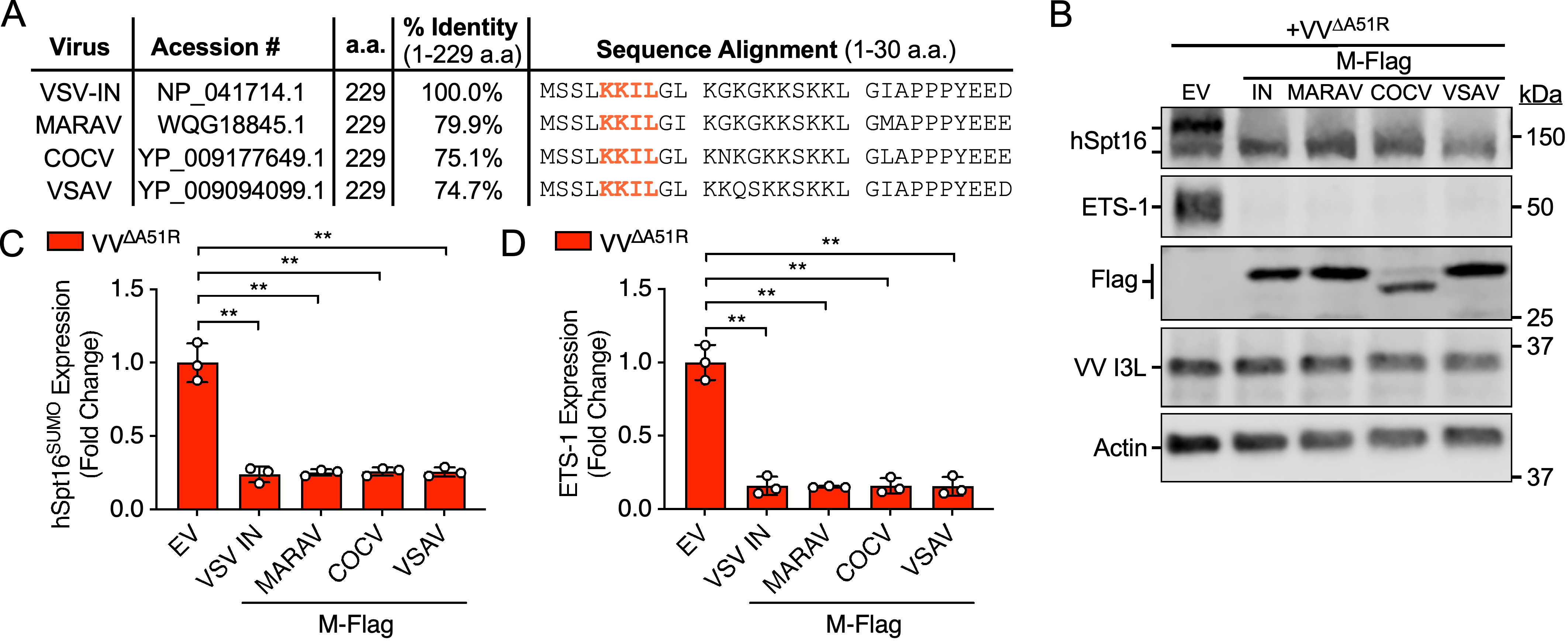

Supplement: S9 Fig — (A) Overall percent amino acid (a.a.) identity of full-length vesiculovirus M proteins relative to VSV M-IN with sequence alignment of the first 30 a.a. of the indicated M proteins highlighting the “KKIL” motif in orange. (B) Representative IB of endogenous hSpt16 and ETS-1 in U2OS WCE following transient transfection with empty vector (EV) or Flag-tagged M proteins from VSV-IN, Maraba virus (MARAV), Cocal virus (COCV), and Alagoas virus (VSAV). Cells were infected with ΔA51R (VVΔA51R; MOI = 10) to induce ETS-1 expression. (C–D) Densitometric quantification of hSpt16SUMO (C) and ETS-1 (D) from multiple independent immunoblot experiments performed as in (B). Data are means ± SD; n = 3. Results of unpaired two-tailed Student’s t-test between protein levels in empty vector (EV) WCE and WCE expressing Flag-tagged proteins are shown above each bar graph as: ** = P < 0.01 or ns = not significant. (TIF) [file ppat.1014430.s009.tif]
